# Suppression of *NtZIP4A*/*B* Changes Zn and Cd Root-to-Shoot Translocation in a Zn/Cd Status-Dependent Manner

**DOI:** 10.3390/ijms22105355

**Published:** 2021-05-19

**Authors:** Karolina Maślińska-Gromadka, Anna Barabasz, Małgorzata Palusińska, Katarzyna Kozak, Danuta Maria Antosiewicz

**Affiliations:** Institute of Experimental Plant Biology and Biotechnology, Faculty of Biology, University of Warsaw, 1 Miecznikowa Str., 02-096 Warszawa, Poland; k.maslinska@student.uw.edu.pl (K.M.-G.); barabasz@biol.uw.edu.pl (A.B.); malgorzata.palusinska@gmail.com (M.P.); katarzyna.kozak@biol.uw.edu.pl (K.K.)

**Keywords:** cadmium, GUS, *NtZIP4*, root-to-shoot translocation, tobacco, zinc, *ZIP* genes, Zinpyr-1

## Abstract

In tobacco, the efficiency of Zn translocation to shoots depends on Zn/Cd status. Previous studies pointed to the specific contribution of root parts in the regulation of this process, as well as the role of *NtZIP4A*/*B* (from the ZIP family; Zrt Irt-like Proteins). Here, to verify this hypothesis, *NtZIP4A*/*B* RNAi lines were generated. Then, in plants exposed to combinations of Zn and Cd concentrations in the medium, the consequences of *NtZIP4A*/*B* suppression for the translocation of both metals were determined. Furthermore, the apical, middle, and basal root parts were examined for accumulation of both metals, for Zn localization (using Zinpyr-1), and for modifications of the expression pattern of *ZIP* genes. Our results confirmed the role of *NtZIP4A*/*B* in the control of Zn/Cd-status-dependent transfer of both metals to shoots. Furthermore, they indicated that the middle and basal root parts contributed to the regulation of this process by acting as a reservoir for excess Zn and Cd. Expression studies identified several candidate *ZIP* genes that interact with *NtZIP4A*/*B* in the root in regulating Zn and Cd translocation to the shoot, primarily *NtZIP1-like* in the basal root part and *NtZIP2* in the middle one.

## 1. Background

The efficiency of zinc (Zn) root-to-shoot translocation is a key factor conditioning the supply of the shoot with this micronutrient needed for the proper development of the plant. In the presence of cadmium (Cd) and also excess Zn in the environment, processes that regulate translocation may limit or increase delivery of these elements to the shoots, impacting the level of accumulation and, consequently, plant-derived food quality or a plant’s industrial use. Zinc and cadmium are chemically similar, and the interaction between these two metals has long been studied [1,2,3,4]. Tobacco is known for high efficiency of Cd and Zn translocation to leaves, which, on one hand, is a danger to smokers [5], but on the other, is an advantage for its use to phytoremediate contaminated soils [6,7]. However, the mechanisms underlying root-to-shoot translocation of Zn and Cd (and also other metals) are not well understood [8,9]. Two major factors are involved in the regulation of the efficiency of metal transfer to aerial plant parts.

The first is the capacity of roots to retain metals, which determines the efficiency of radial transport and ultimately the amount available for upward translocation. It mainly depends on metal sequestration in the vacuoles. For example, it has been shown that in the Zn-hyperaccumulating ecotype of *Sedum alfredii*, there are 2.7-times less Zn in the root vacuoles relative to the nonaccumulating type [10]. Similar results were obtained in comparative studies on Zn and Cd hyperaccumulator *Thlaspi caerulescens* (current name *Noccaea caerulescens*) and nonaccumulating *T. arvense* [11]. Furthermore, in their study on variation among different accessions of *T. caerulescens* in root-to-shoot translocation of Zn and Cd, Xing et al. [12] found that higher translocation efficiency was associated with a lower level of metal accumulation in root vacuoles. Similarly, research on rice has shown that OsHMA3 localized in the tonoplast is responsible for loading Cd into the root vacuoles, and thus consequently for low cadmium translocation to shoots, which led to low Cd accumulation in rice grains [13,14].

The second is loading of metals into xylem vessels. The importance of xylem loading in efficient translocation of metals has been reported in several studies. It was found, for example, to be a critical factor in efficient Cd accumulation in the shoots of *Solanum* species [15] and also in high Cd accumulation varieties of rice or *Sedum alfredii* [16,17]. Several studies led to identification of *HMA4* as a key gene responsible for this process in *A. thaliana*, *A. halleri*, *T. caerulescens*, or *N. tabacum* [18,19,20,21,22] and for the regulation of the rate of Zn and Cd translocation to shoots. It encodes a plasma membrane localized P_IB_-type Heavy Metal ATPase that transports Zn and Cd to the apoplast. Being expressed in the root’s vasculature, it contributes to the loading of both metals into the apoplast of xylem vessels. The level of expression, which depends on copy numbers and cis-regulatory elements, determines the efficiency of loading metals into xylem vessels and thus the translocation [21].

Additionally, it has been shown that the efficiency of root-to-shoot translocation of metals depends on the concentration of a given metal and on the mineral composition of the medium. For example, the dependence of Zn translocation efficiency on external Zn has been shown in *A. thaliana* [23] and in tobacco [24,25]. Furthermore, Zn translocation depends on the presence of other metals in the medium, including Cd. Inhibition of translocation has often been described [26,27,28]; however, our recent study showed stimulation in the presence of certain Cd concentrations [28,29]. An increase in Zn translocation was found in the presence of 0.25 and 1 μM Cd in plants grown at low/medium Zn concentrations (0 to 1 μM). To compare, for plants exposed to 5–50 μM Zn, translocation of Zn to shoots was reduced in the presence of 4 μM Cd (relative to the medium without Cd).

Roots are the organs that determine the effectiveness of metal/s translocation. Their structure is complex, with different meristematic, primary, and secondary tissues. However, the vast majority of research aimed at identifying processes involved in the regulation of metals transfer to shoots (primarily molecular processes and accumulation) has been carried on whole roots.

Our recent study on tobacco showed that at exposure to distinct combinations of Zn/Cd concentrations in the medium, the apical, middle, and basal root parts differ physiologically and molecularly. Their different capacities to accumulate both metals that depended on Zn/Cd status were revealed, and the middle and basal parts were suggested to play a role of a sink for excess Zn/Cd in the medium. Moreover, of the nine *ZIP* genes (Zrt Irt-like Proteins); [30] tested, for eight (*NtZIP1-like, NtZIP2, NtZIP4A*/*B, NtZIP5A*/*B, NtZIP5-like, NtZIP8, NtZIP11, NtIRT1*, and *NtIRT1-like*), the expression pattern was specific to the root part [28]. Altogether, these results were the first to indicate the distinct role of the root parts in the regulation of Zn/Cd status-dependent translocation efficiency.

Of the *ZIP* genes tested in tobacco, only one, *NtZIP4A*/*B*, upregulated by Zn deficiency, showed similar transcript levels in all parts of the root at tested combinations of Zn/Cd concentrations [28]. There are two copies of *NtZIP4*, *NtZIP4A*, and *NtZIP4B* (sharing 97.57% homology at the amino acid level) with the same expression pattern in tobacco [31]. *NtZIP4B* encodes a Zn and Cd uptake protein. GUS-based analysis of the spatial expression indicated involvement of NtZIP4B in the uptake of both metals by the middle root part directly from the soil solution (GUS staining in the epidermis), but also in other root tissues, suggesting a role in delivery of metals to the cells of the internal tissues [31].

The aim of this study was to add to the knowledge about the physiological role of *NtZIP4* in tobacco, especially about the previously hypothesized contribution to the regulation of Zn/Cd-status dependent translocation of both metals to shoots [29]. We generated *NtZIP4A*/*B*-RNAi lines, and conducted detailed analysis of silencing consequences for Zn/Cd translocation, metal accumulation in parts of the root, and analysis of accompanying expression modifications of other *ZIP* genes. The presented data indicate a role of *NtZIP4A*/*B* in the translocation of Zn and Cd, but only at specific combinations of Zn/Cd concentrations in the medium.

## 2. Results

### 2.1. Generation and General Characteristics of NtZIP4A/B-RNAi Tobacco Plants

An overall description of the generation and characterization of transgenic tobacco *NtZIP4A*/*B*-RNAi is given in Supplementary Figure S1. In total, twenty eight transgenic heterozygous T1 lines were obtained, of which twelve displayed a segregation ratio of 3:1 (tolerant^kan^:sensitive^kan^) suggesting one insert location. Out of those, eight homozygous T2 lines were developed (numbers 3, 4, 6, 7, 8, 9, 10, and 11).

The reduction of the expression level of both *NtZIP4A* and *NtZIP4B* was shown in Supplementary Figure S1c. Knowing that *NtZIP4A* and *NtZIP4B* are upregulated at Zn deficiency [31], to determine the degree of transcript reduction, tested RNAi lines and WT plants were grown in Zn deficient conditions for four days and in the control medium. Expression decreased to varying degrees in the RNAi lines. The reduction in the range of 20% to 55% of the wild-type level was found in five lines (numbers 4, 6, 7, 8, and 9); however, it remained unchanged in three lines (numbers 3, 10, and 11). Four lines (numbers 4, 6, 7, and 8) with the highest reduction of the *NtZIP4A*/*B* expression were further grown in soil until seed. Their height, number of leaves, and flowers remained at the level of the reference wild-type (Supplementary Figure S1d).

For further experiments two lines differing in the level of *NtZIP4A*/*B* suppression were selected (numbers 4 and 6). For the line number 4, the reduction of *NtZIP4A*/*B* transcript abundance in the Zn-deficient roots was 42% of the wild-type, while it was 18% for the line number 6 (Supplementary Figure S1c).

### 2.2. Suppression of NtZIP4A/B Decreased Efficiency of Zn/Cd Status-Dependent Zn and Cd Translocation of Both Metals to Shoots

**Figure 1:**
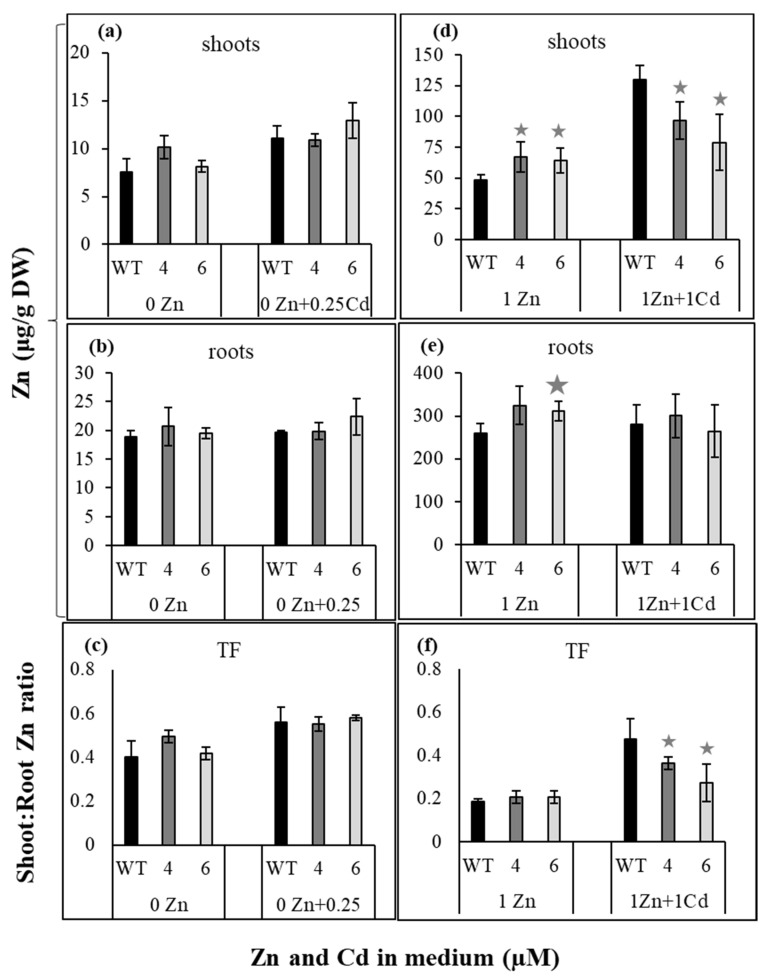
Zinc concentration in wild-type and *NtZIP4A*/*B*-RNAi plants, grown under various Zn and Cd concentrations. 3.5-week-old plants of wild-type (WT) and *NtZIP4A*/*B*-RNAi lines (number 4 and number 6) grown in the quarter-strength Knop’s medium were exposed for 17 days to the control medium supplemented with pairwise combinations of Zn (0; 1 μM) and Cd (0; 0.25; 1 μM) concentrations. Concentrations of zinc were determined in the shoots (**a**,**d**) and roots (**b**,**e**). The Translocation Factor (TF) was determined as the ratio of shoot/root concentrations of Zn (**c**,**f**). Values correspond to means ± SD (*n* = 5); those significantly different from wild-type (WT) (evaluated by Student’s *t*-test) are indicated by asterisks 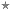 (*p* ≤ 0.05).

**Figure 2:**
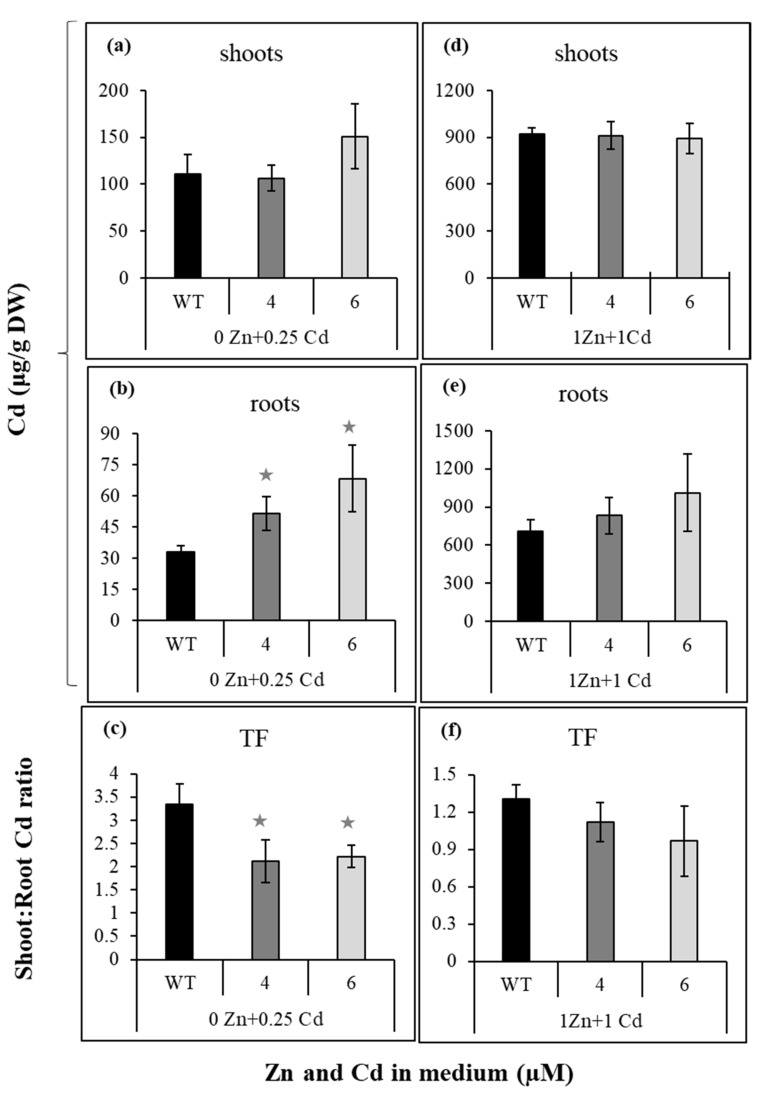
Cadmium concentration in wild-type and *NtZIP4A*/*B*-RNAi plants grown under various Zn and Cd concentrations. 3.5-week-old plants of wild-type (WT) and *NtZIP4A*/*B*-RNAi lines (number 4 and number 6) grown in the quarter-strength Knop’s medium were exposed for 17 days to the control medium supplemented with pairwise combinations of Zn (0; 1 μM) and Cd (0; 0.25; 1 μM) concentrations. Concentrations of cadmium were determined in the shoots (**a**,**d**), and roots (**b**,**e**). The Translocation Factor (TF) was determined as the ratio of shoot/root concentrations of Cd (**c**,**f**). Values correspond to means ± SD (*n* = 5); those significantly different from wild-type (WT) (evaluated by Student’s *t*-test) are indicated by asterisks 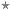 (*p* ≤ 0.05).

Previous studies showed that Zn translocation to shoots was stimulated in the presence of Cd [28,29]. To determine whether suppression of *NtZIP4A*/*B* affects the efficiency of Zn and Cd translocation to shoots, plants were exposed to the basal ¼ Knop’s medium supplemented with combinations of Zn (0 Zn; 1 µM Zn) and Cd (0; 0.25; 1 µM) concentrations (they were chosen based on previous studies [28]). Concentrations of Zn and Cd in the shoots, roots, and Transloction Factor were shown in Figure 1 and Figure 2. Of importance was finding that suppression of *NtZIP4A*/*B* affected the efficiency of Zn and Cd translocation to shoots in a Zn/Cd concentration-dependent manner. It reduced Zn translocation to shoots, but only in plants exposed to 1 µM Zn + 1 µM Cd (Figure 1f). On the other hand, significantly lower Cd Translocation Factor was noted in RNAi plants exposed to 0.25 µM Cd added to the Zn-deficient medium (Figure 2c), and Cd concentration in the roots increased (Figure 2b). At exposure to 1 µM Zn + 1 µM Cd, concentrations of Cd in the roots (Figure 2e) and Translocation Factor (Figure 2f) showed only a tendency to lower, but the difference was not statistically significant. Overall, results indicate that *NtZIP4A*/*B* is involved in the regulation of Zn and Cd translocation to the shoot, which depends on the combination of Zn and Cd concentrations in the medium.

### 2.3. Changes in the Accumulation of Zn and Cd in the Root Parts of NtZIP4A/B-RNAi Plants

**Figure 3:**
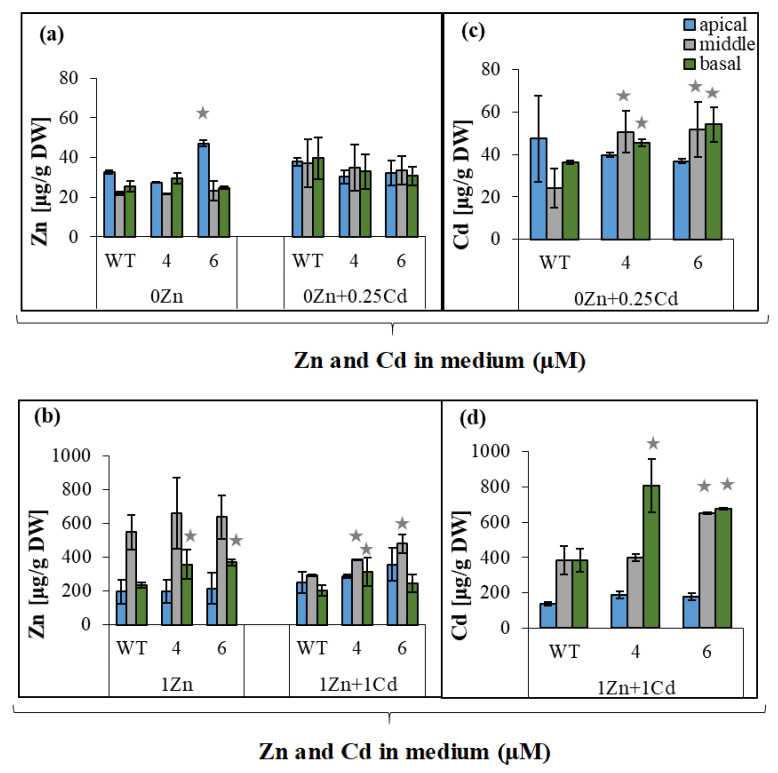
Zinc and cadmium concentration in the apical, middle, and basal parts of the roots from wild-type and *NtZIP4A*/*B*-RNAi plants, grown under various Zn and Cd concentrations. 3.5-week-old plants of wild-type (WT) and *NtZIP4A*/*B*-RNAi lines (number 4 and number 6) grown in the quarter-strength Knop’s medium were exposed for 17 days to the control medium supplemented with pairwise combinations of Zn (0; 1 μM) and Cd (0; 0.25; 1 μM) concentrations. Concentrations of metals were measured in the apical parts (2.5 cm) from the main and lateral roots; basal (proximal) parts (1/4 of the total length of the main roots); middle part (remaining parts of the main roots). The basal and the middle parts of the roots were collected from the main root only (adventitious roots were excised). Zinc concentration (**a**,**b**), cadmium concentration (**c**,**d**). Values correspond to arithmetic means ± SD (*n* = 3); those significantly different from wild-type (WT) (evaluated by Student’s *t*-test) are indicated by asterisks 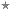 (*p* ≤ 0.05).

Knowing that suppression of *NtZIP4A*/*B* leads to Zn/Cd status-dependent reduction in Zn and Cd translocation (Figure 1 and Figure 2), we then examined if the distribution of Zn and Cd along the root (apical, middle, and basal root parts) was affected in the *NtZIP4A*/*B*-RNAi plants. At Zn deficient medium, with or without 0.25 µM Cd, suppression did not significantly modify (with one exception) concentrations of Zn in the root parts (Figure 3a). Changes were found at higher Zn level in the medium. At 1 µM Zn, more metal was found in the basal root parts of RNAi plants (relative to the wild-type) and in the middle parts after adding 1 µM Cd (Figure 3b).

Reduced expression of *NtZIP4A*/*B* also altered Cd partitioning between the root parts. Concentrations of Cd in the middle and basal root parts were higher than in the wild-type at both experimental variants (except line number 4 at 1 µM Zn + 1 µM Cd) (Figure 3c,d).

### 2.4. Zinpyr-1 Based Determination of Zn Localization in the Root Parts

Next, we used Zinpyr-1 (Zn indicator) to determine if the reduction of the expression of *NtZIP4A*/*B* changes Zn localization in the apical, middle, and basal root parts of plants exposed to the tested four variants of medium composition.

At the Zn-deficient medium (with or without 0.25 µM Cd), the intensity of green fluorescence indicating Zn localization was very low, close to the level of autofluorescence. Therefore, it was not conclusive as to possible differences between experimental variants (Supplementary Figure S2).

**Figure 4:**
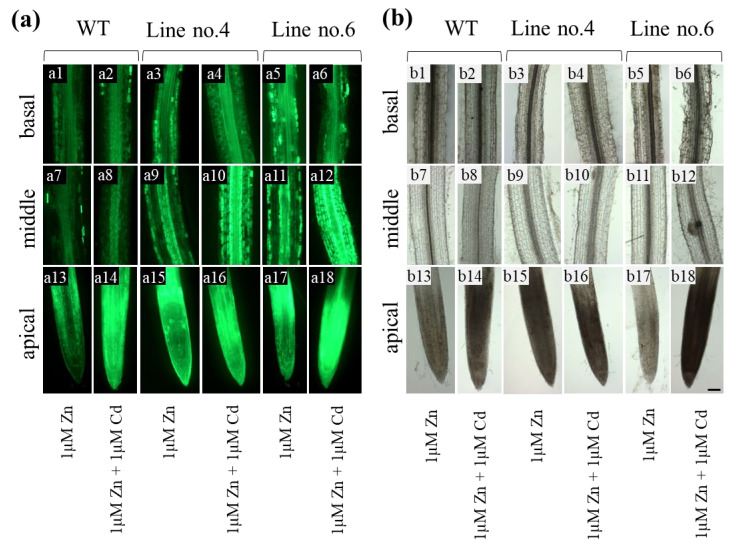
Zn distribution at the longitudinal sections through the apical, middle, and basal parts of the roots from wild-type and *NtZIP4A*/*B*-RNAi plants, grown under various Zn and Cd concentrations, visualized by Zinpyr-1. 3.5-week-old plants of wild-type (WT) and *NtZIP4A*/*B*-RNAi lines (number 4 and number 6) grown in the quarter-strength Knop’s medium were exposed for 17 days to the control medium supplemented with 1 μM Zn with or without 1 μM Cd. Fluorescence microscope images of longitudinal sections through the apical, middle, and basal root parts (**a**). Representative pictures of the anatomical structure of the root parts at bright field (**b**). Plants grown at 1 μM Zn (**a1**,**a7**,**a13**,**a3**,**a9**,**a15**,**a5**,**a11**,**a17**,**b1**,**b7**,**b13**,**b3**,**b9**,**b15**,**b5**,**b11**,**b17**); at 1 μM Zn + 1 μM Cd (**a2**,**a8**,**a14**,**a4**,**a10**,**a16**,**a6**,**a12**,**a18**,**b2**,**b8**,**b14**,**b4**,**b10**,**b16**,**b6**,**b12**,**b18**). Wild-type plants (WT); RNAi plant lines (number 4 and number 6). Magnification bars = 0.25 mm.

The Zn localization in the roots grown on the medium containing 1 µM Zn (with or without 1 µM Cd) was shown in Figure 4a, and sections at bright field at Figure 4b. In the apical parts of the wild-type plants exposed to 1 µM Zn, the strongest green signal was found above the quiescent center (QC) in the differentiating cortex cells (Figure 4(a13)). After adding 1 µM Cd, the intensity of green signal in the whole root apex increased (Figure 4(a14)). In the middle and basal root parts of wild-type plants Zn distribution was not significantly different between treatments (Figure 4(a7 vs. a8); Figure 4(a1 vs. a2)). Overall, a higher amount of Zn was present in the stele, lower in the cortex. Interestingly, moderately higher accumulation was detected in the cortex of the basal root segment.

Suppression of *NtZIP4A*/*B* changed the localization of Zn qualitatively and quantitatively. In general, in RNAi lines, the green fluorescence in all root parts was stronger than in the wild-type, especially in line number 6 (Figure 4(a3–a6,a9–a12,a15–a18)). In the apical parts of the roots exposed to 1 µM Zn, more Zn was found above QC in the differentiation zone (Figure 4(a15,a17)), and the intensity of fluorescence increased in the presence of 1 µM Cd (Figure 4(a16,a18)). In the middle root parts, a high amount of Zn was detected in numerous single cells within the epidermis and the first cortex layer and at the border between the cortex and stele (Figure 4(a9,a11 vs. a10,a12)). The level of Zn accumulation slightly increased in the presence of 1 µM Cd. In the basal root parts, Zn was accumulated primarily in the cortex cells but not in the epidermis (Figure 4(a3–a6)).

### 2.5. Spatial Expression Pattern of NtZIP4B in the Root Parts Depends on the Combinations of Zn/Cd Concentrations in the Medium

**Figure 5:**
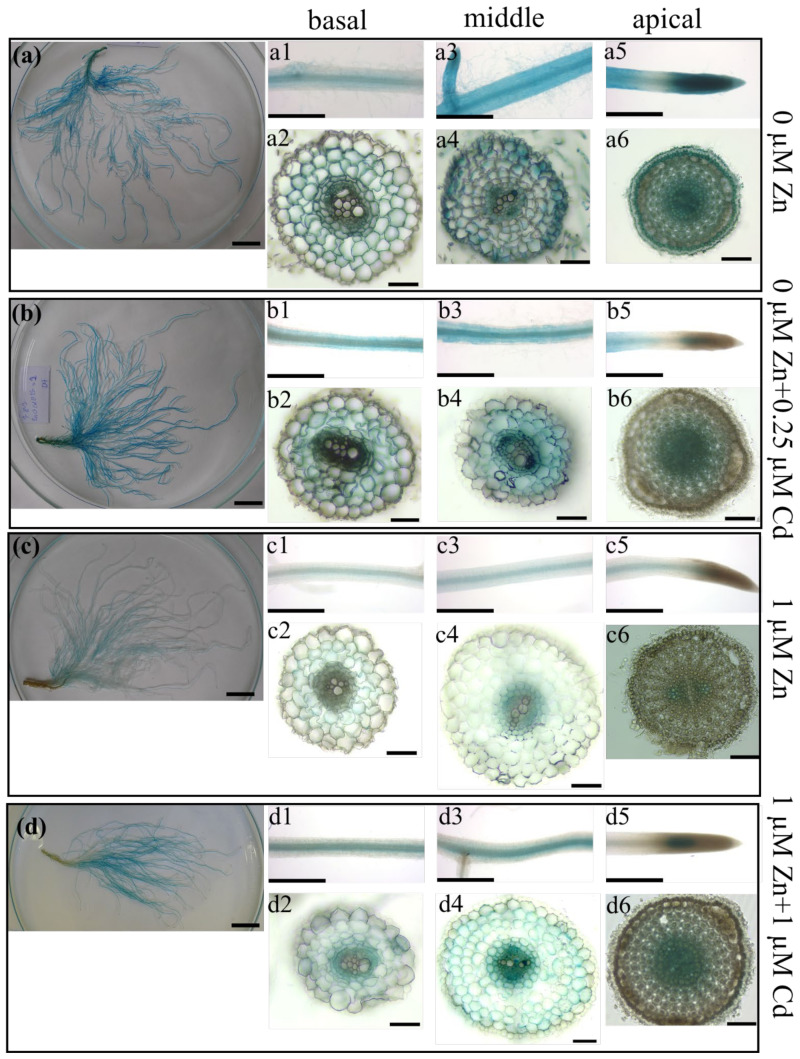
GUS staining pattern of transgenic plants expressing *NtZIP4B_prom_*::*GUS*. GUS expression in five-week old transgenic plants expressing *NtZIP4B_prom_*::*GUS* grown in the quarter-strength Knop’s medium for 3.5 weeks and subsequently exposed for 17 days to the control medium supplemented with pairwise combinations of Zn (0; 1 μM) and Cd (0; 0.25; 1 μM) concentrations. Whole roots (**a**–**d**). Basal root parts (**a1**,**b1**,**c1**,**d1**) and the cross sections (**a2**,**b2**,**c2**,**d2**). Middle root parts (**a3**,**b3**,**c3**,**d3**) and the cross sections (**a4**,**b4**,**c4**,**d4**). Apical root parts (**a5**,**b5**,**c5**,**d5**) and the cross sections (**a6**,**b6**,**c6**,**d6**). Magnification bars: 2 cm for (**a**–**d**), 1 mm for (**a1**,**a3**,**a5**,**b1**,**b3**,**b5**,**c1**,**c3**,**c5**,**d1**,**d3**,**d5**); 0.1 mm for (**a2**,**a4**,**a6**,**b2**,**b4**,**b6**,**c2**,**c4**,**c6**,**d2**,**d4**,**d6**).

In our previous study [31], the spatial expression pattern of *NtZIP4Bp::GUS* has been determined in plants grown at Zn deficiency and at control ¼ Knop’s medium. Here, to learn more about the physiological role of NtZIP4B, the same transgenic lines were used to investigate whether the *NtZIP4B* promoter activity in the root parts depended on Zn/Cd status (Figure 5).

Overall, the level of *NtZIP4Bp*::GUS expression was high in the Zn-deficient roots (Figure 5a) and decreased with higher Zn concentration (1 µM) (Figure 5c). Furthermore, the addition of Cd to the nutrient solution (both to Zn deficient medium and containing 1 µM Zn) resulted in an increase in the promoter activity (Figure 5b,d). The spatial pattern of *NtZIP4Bp*::GUS expression also depended on Zn/Cd status. In the apical parts of Zn-deficient roots, strong GUS-dependent blue staining was present in the root cap and meristematic tissues close to the quiescent center (QC), with the highest intensity in the procambium (Figure 5(a5,6)). However, promoter activity in these tissues was lost at higher 1 µM Zn (Figure 5(c5,c6)), and in the presence of Cd (0 Zn + 0.25 µM Cd; 1 µM Zn + 1 µM Cd) (Figure 5(b5,b6),(d5,d6)), respectively. GUS-dependent blue staining was detected further up from the QC in the differentiating procambial cells and to a lesser extent in the cortex cells (Figure 5(b5,b6),(c5,c6),(d5,d6)).

In the middle parts of the roots exposed to Zn deficiency, intense blue staining was seen in the epidermis and in the vascular tissue, and much weaker in the cortex cells (Figure 5(a3,a4)). However, promoter activity was not detected in the epidermis in the presence of higher 1 µM Zn (Figure 5(c3,c4)) and in the presence of 0.25 and 1 µM Cd (Figure 5(b3,b4),(d3,d4)). In the cortex, GUS activity decreased to almost undetectable level when Zn concentration in the medium went up to 1 µM Zn (Figure 5c(c3,c4)), but increased in the presence of Cd (Figure 5(d3,d4)).

In the basal root parts, no promoter activity was found in the epidermis in any of the experimental variants; however, it was detected in other tissues (Figure 5(a1,a2),(b1,b2),(c1,c2),(d1,d2)), and changes that depended on the Zn/Cd status were similar to those seen in the middle part (Figure 5(a3,a4),(b3,b4),(c3,c4),(d3,d4)). Briefly, results indicate that the uptake of Zn and Cd mediated by NtZIP4B is highly reduced at 1 µM Zn and in the presence of Cd, compared to the Zn-deficiency conditions. It is worth noting that apart from the reduced activity of the *NtZIP4B* promoter in the internal tissues of the root, it disappears in the epidermis of the central parts of the root at 1 µM Zn (with or without Cd), which likely leads to a lower influx of toxic Cd and also excess Zn.

### 2.6. Zn/Cd Status-Dependent Expression of Tobacco ZIP Genes in the Root Parts of NtZIP4A/B-RNAi Plants

Further, the aim was to investigate the role of *NtZIP4A*/*B* in the regulation of Zn/Cd status-dependent translocation of both metals to the shoots and learn more about a specific role of the root parts in this process. We investigated if the expression of *NtZIP4A*/*B* in the apical, middle, and basal root parts depends on Zn and Cd concentrations in the medium, and to what extent suppression of *NtZIP4A*/*B* affects the root-part-specific transcript abundance of tobacco *ZIP* genes. Stability of the reference gene *PP2A* was shown in Supplementary Figure S3.

**Figure 6:**
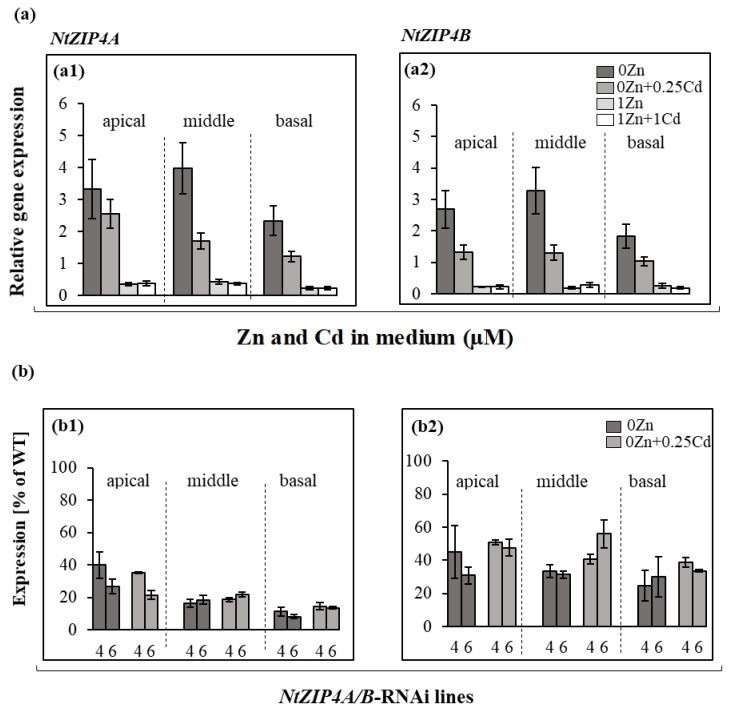
*NtZIP4A and NtZIP4B* transcript levels in the root parts of wild-type and *NtZIP4A*/*B*-RNAi plants grown at various Zn and Cd concentrations. 3.5-week-old plants of wild-type (WT) and *NtZIP4A*/*B*-RNAi lines (number 4 and number 6) grown in the quarter-strength Knop’s medium were exposed for 17 days to the control medium supplemented with pairwise combinations of Zn (0; 1 μM) and Cd (0; 0.25; 1 μM) concentrations. Transcript levels were monitored by RT-qPCR in the apical, middle, and basal root parts (**a**). Expression in the wild-type plants of *NtZIP4A* (**a1**) and *NtZIP4B* (**a2**). Expression of *NtZIP4A* (**b1**) and *NtZIP4B* (**b2**) in the *NtZIP4A*/*B*-RNAi plants were shown as a percentage of expression of the wild-type in the same part of the root grown at the same medium composition (**b**). The transcript levels of *NtZIP4A* and *NtZIP4B* were normalized to *PP2A* level. Values correspond to means ±SD (*n* = 3); those with a ratio greater than 2 are considered significantly different (marked by an asterisk) [32].

In the wild-type plants, both *NtZIP4A* and *NtZIP4B* responded similarly to applied growth conditions in the apical, middle, and basal root parts (Figure 6(a1,a2)). The highest expression was found at Zn-deficiency, and was moderately reduced in the presence of 0.25 µM Cd. Upon exposure to 1 µM Zn, the amount of transcript highly decreased (to a similar extent in the presence or absence of Cd).

The level of reduction of *NtZIP4A*/*B* expression in the RNAi plants was in the range of 40–50% of the expression of the reference wild-type in all tested root parts (Figure 6(b1,b2)). Results were shown only for Zn-deficient medium, as in RNAi plants the expression was very low in the presence of 1 µM Zn (data not shown).

**Figure 7:**
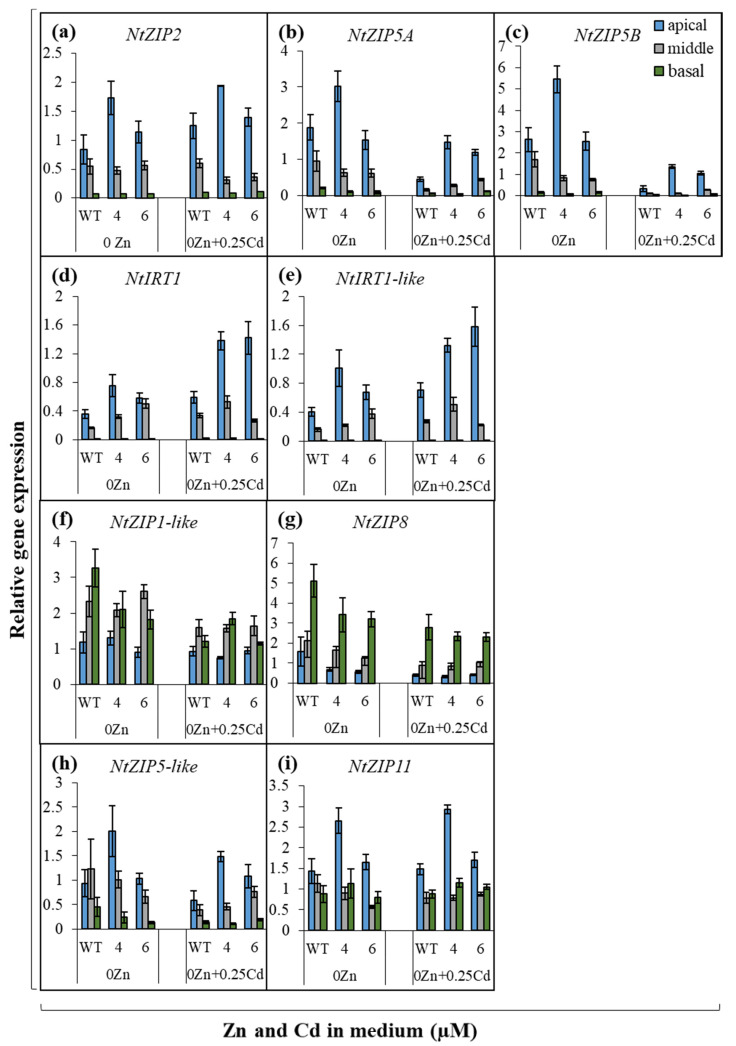
Expression of tobacco *ZIP* genes in the apical, middle, and basal parts of wild-type and *NtZIP4A*/*B*-RNAi plants grown at Zn deficient medium with or without 0.25 μM Cd. 3.5-week-old plants of wild-type (WT) and *NtZIP4A*/*B*-RNAi lines (number 4 and number 6) grown in the quarter-strength Knop’s medium were exposed for 17 days to the Zn-deficient medium (Zn was not added to the medium) with or without 0.25 μM Cd. Expression of *NtZIP2* (**a**), *NtZIP5A* (**b**), *NtZIP5B* (**c**), *NtIRT1* (**d**), *NtIRT1-like* (**e**), *NtZIP1-like* (**f**), *NtZIP8* (**g**), *NtZIP5-like* (**h**), and *NtZIP11* (**i**). Gene expression was normalized to the *PP2A* level. Values correspond to arithmetic means ± SD (*n* = 3); those with a ratio greater than 2 are considered significantly different [32].

**Figure 8:**
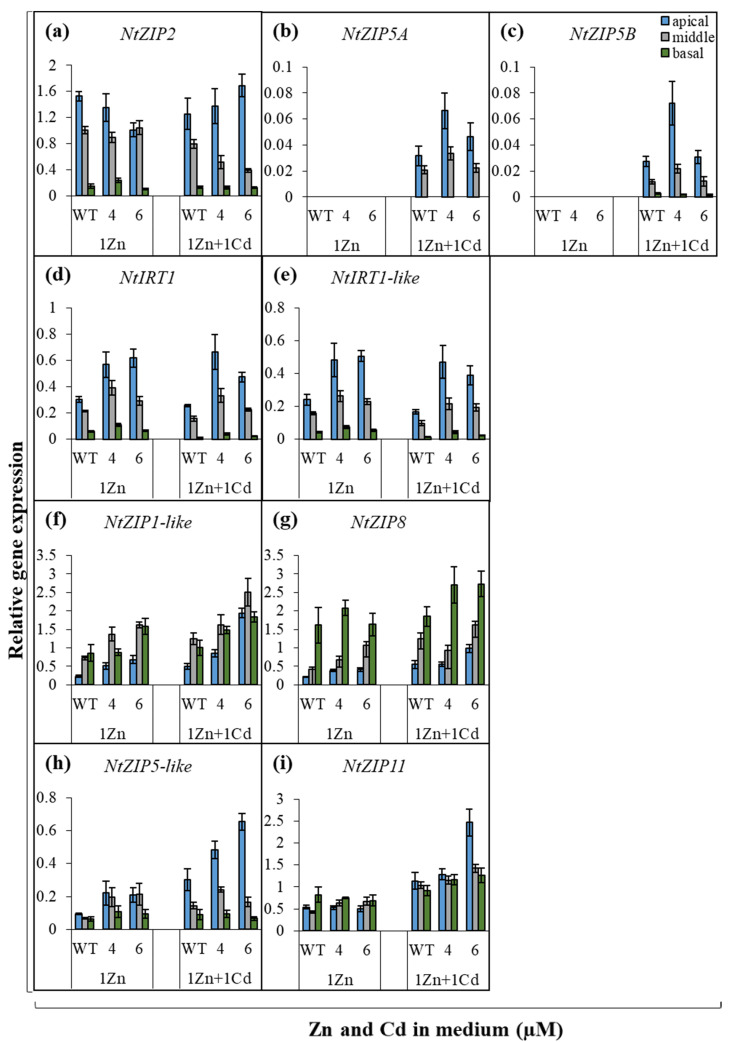
Expression of tobacco *ZIP* genes in the apical, middle, and basal parts of wild-type and *NtZIP4A*/*B*-RNAi plants grown at 1 μM Zn with or without 1 μM Cd. 3.5-week-old plants of wild-type (WT) and *NtZIP4A*/*B*-RNAi lines (number 4, number 6) grown in the quarter-strength Knop’s medium were exposed for 17 days to the control medium supplemented with 1 μM Zn with or without 1 μM Cd. Expression of *NtZIP2* (**a**), *NtZIP5A* (**b**), *NtZIP5B* (**c**), *NtIRT1* (**d**), *NtIRT1-like* (**e**), *NtZIP1-like* (**f**), *NtZIP8* (**g**), *NtZIP5-like* (**h**), and *NtZIP11* (**i**). Gene expression was normalized to the *PP2A* level. Values correspond to arithmetic means ± SD (*n* = 3); those with a ratio greater than 2 are considered significantly different [32].

Experiments were then performed to determine whether suppression of *NtZIP4A*/*B* modifies the expression of *ZIP* genes in the root parts (Figure 7 and Figure 8). Significant changes in the transcript levels of some of the analyzed genes were detected, and the pattern depended on the concentrations of Zn and Cd in the medium and on the root part.

Out of nine examined *ZIP* genes (*NtZIP1-like, NtZIP2, NtZIP4A*/*B, NtZIP5A*/*B, NtZIP5-like, NtZIP8, NtZIP11, NtIRT1, NtIRT1-like*), the greatest changes were found in the apical root parts. First, *NtIRT1* and *NtIRT1-like* were upregulated at all four tested variants of Zn/Cd concentrations in the medium (0Zn, 0Zn + 0.25 µM Cd, 1 µM Zn, 1 µM Zn + 1 µM Cd) (Figure 7d,e and Figure 8d,e). Second, at 1 µM Zn, transcript levels of *NtZIP1-like* and *NtZIP5-like* were higher regardless of the presence of 1 µM Cd (Figure 8f,h). Furthermore, Zn/Cd-concentration-dependent response was found for *NtZIP5A*/*B* in the apical segment (upregulation at 0Zn + 0.25 µM Cd; Figure 7b,c) and for *NtZIP8* (downregulation at 0 Zn; Figure 7g).

In the middle root parts of RNAi plants, fewer *ZIP* genes had altered expression pattern relative to the wild-type, and responses were less general under the experimental conditions used. At Zn, deficiency-increased transcript abundance was found for *NtIRT1* (Figure 7d), while at 1 µM Zn for *NtZIP1-like* and *NtZIP5-like* (Figure 8f,h). Furthermore, *NtZIP2* was downregulated specifically in the presence of Cd, regardless of the Zn concentration (Figure 7a and Figure 8a). In the basal root parts of *NtZIP4A*/*B*-RNAi plants, only one gene with modified expression pattern was identified. It was *NtZIP1-like*, whose expression was higher in both transgenic lines (compared to the wild-type) when grown on medium containing 1 µM Zn + 1 µM Cd, but at 1 µM Zn in line number 6 only (Figure 8f).

Obtained results indicate a significant role of *ZIP* genes in generating the phenotype of tobacco with the suppression of *NtZIP4A*/*B*. Of importance is detected root-part-specific and Zn/Cd-status-dependent up and downregulation, which likely contribute to changes in the Zn accumulation efficiency and to changes in the Zn localization pattern.

*NtHMA4α*/*β* is known to control the efficiency of Zn and Cd translocation to shoots [22]. Therefore, in addition to tobacco *ZIP* genes, we determined its expression in the root parts and showed that it was not different between the wild-type and RNAi plants (Supplementary Figure S4). It was only higher at 1 µM Zn in all tested lines as compared with Zn-deficiency conditions.

### 2.7. ^65^Zn Based Analysis of Zn Translocation to Shoots

**Figure 9:**
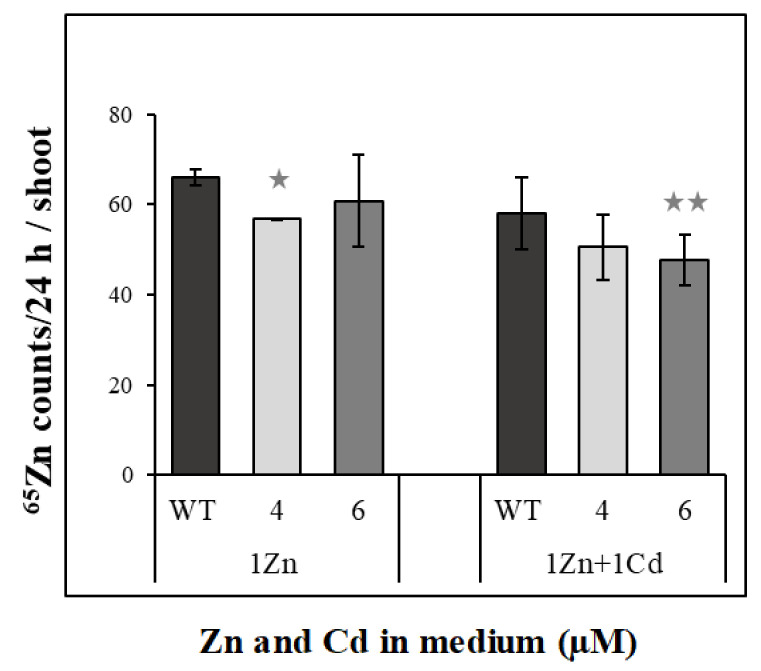
Total ^65^Zn counts/24h in the shoots of wild-type and *NtZIP4A*/*B*-RNAi plants. 3.5-week-old plants of wild-type (WT) and *NtZIP4A*/*B*-RNAi lines (number 4 and number 6) grown in the quarter-strength Knop’s medium were exposed for six days to the control medium supplemented with 1 μM Zn with or without 1 μM Cd. For the seventh day (the last day of the experiment), seedlings were transferred to the Petri dishes filled with the medium of the same composition solidified with agar and covered with a thin layer of the liquid medium. The basal root part was immersed in the small vial filled with the medium of the same composition containing ^65^Zn. Values correspond to means ± SD (*n* = 3); those significantly different from wild-type (WT) (evaluated by Student’s *t*-test) are indicated by one asterisk 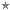 (*p* < 0.05) and two asterisks 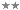 (*p* < 0.01).

Previous experiments suggested possible contribution of *NtZIP4A*/*B* to the regulation of Zn/Cd status-dependent translocation to shoots of both metals, and the involvement of the basal root part by retaining excess metal. To learn more, here we applied ^65^Zn to the basal root parts of the wild-type and RNAi plants and determined the amount of radioactive Zn transferred to shoots. Suppression of *NtZIP4A*/*B* resulted in a moderate reduction of the amount of ^65^Zn in the shoots of RNAi plants; however, the difference was statistically significant for one line only (Figure 9).

## 3. Discussion

Our previous research showed that the effectiveness of Zn and Cd root-to-shoot translocation depends on mutual concentrations of both metals in the medium [28,29]. Here, RNA interference (RNAi) plants with reduced expression of *NtZIP4A*/*B* were generated to investigate its possible involvement in the regulation of this phenomenon. Experiments were performed on the combinations of Zn and Cd concentrations (0 Zn; 0 Zn + 0.25 µM Cd; 1 µM Zn; 1 µM Zn + 1 µM Cd) used previously in our study on Zn/Cd status-dependent Zn and Cd root-to-shoot translocation [28].

Interestingly, reduction of the expression of *NtZIP4A*/*B* (Figure 6(b1,b2)) resulted in decreased translocation of both Zn and Cd, however for each metal at different combinations of Zn/Cd concentrations in the medium (Figure 1 and Figure 2). This indicates the involvement of *NtZIP4A*/*B* in regulating Zn/Cd translocation, but also the existence of distinct mechanisms, very specific for a given concentration of both metals, that contribute to diversified responses. Such changes are likely accompanied by modifications of the expression of other metal homeostasis genes. Here, we investigated the possible involvement of previously characterized tobacco *ZIP* genes [28,29,31].

### 3.1. Suppression of NtZIP4A/B Decreased Zn Translocation to Shoots at 1 µM Zn + 1 µM Cd

Tests on RNAi plants performed on two variants of Zn/Cd concentrations in the medium (0 Zn + 0.25 µM Cd; 1 µM Zn + 1 µM Cd) showed that suppression of *NtZIP4A*/*B* reduced the efficiency of Zn translocation only in plants grown at 1 µM Zn + 1 µM Cd (Figure 1f). It is known that the ability of the root to accumulate Zn is one of the key factors determining the efficiency of translocation [15,33,34]. For example, *OsZIP4* was implicated in the regulation of Zn distribution between the root and shoot [35]. Its overexpression under the 35S promoter decreased translocation efficiency, leading to Zn-deficiency in the shoots. The literature does not provide the results of studies quantifying the contribution of root sectors to Zn translocation to date. Our recent research suggested that the middle and basal root parts, by their ability to accumulate excess metals (at 1 µM Zn + 1 µM Cd), have a specific role in the regulation of Zn/Cd-status-dependent translocation to shoots of both metals [28]. Therefore, it was of importance to know whether longitudinal root Zn distribution changes in RNAi plants in a Zn/Cd status-dependent manner, as well as to determine the Zn tissue-specific localization in the analyzed parts of the root. We found that in plants with a reduced *NtZIP4A*/*B* transcript level, Zn was retained in the middle and basal root parts more efficiently than in the wild-type (Figure 3b). Interestingly, this was accompanied by modified Zn localization (Figure 4). In the middle part of the roots from RNAi plants, the highest Zn level was found in the subepidermal cortex layer, in the individual epidermal cells, and also on the border between the cortex and the stele (Figure 4(a9–a12)), while in the basal part primarily in the cortex (Figure 4(a3,a6)).

GUS-based histochemical analysis showed *NtZIP4B* promoter activity in the vasculature and cortex of both root parts, but it disappeared from the epidermis of the middle part at 1 µM Zn regardless of Cd presence (Figure 5(b3,b4),(c3,c4),(d3,d4)). Thus, the NtZIP4 uptake transporter provides cells with Zn and also toxic Cd. The metal taken up by a cell can be stored in vacuoles, but can be also transferred radially towards the central cylinder by a symplastic pathway [36]. Thus, the expression level of *NtZIP4A*/*B* may determine the efficiency of the symplastic radial transport of Zn and Cd towards xylem. Consequently, its reduced expression in RNAi plants may induce an increase in the metal pool in the apoplast; however, it can also limit transport via the endodermal barrier to the vascular tissue. The observed accumulation of Zn on the border with the stele (Figure 4(a9–a12)) is in line with this suggestion. Such a mechanism may be responsible for the reduction of Zn translocation efficiency to the shoot at 1 µM Zn + 1 µM Cd (Figure 1f) in *NtZIP4A*/*B*-RNAi plants.

The ionic balance between the cellular compartments is maintained through the synchronized activity of metal homeostasis genes [9,37,38]. For example, functional redundancy among *ZIP* genes in *Arabidopsis* was suggested to account for the lack of detectable phenotypes in *zip1* and *zip2* mutants [39]. In this study, it is thought most likely that suppression of *NtZIP4A*/*B* disturbs metal homeostasis and changes the tissue Zn/Cd status that modifies the expression pattern of other metal transporters to restore balance. Consequently, these processes may also contribute to changes in Zn accumulation and distribution and to a reduction of Zn translocation to shoots of RNAi plants. To learn more about the molecular background, the expression of *ZIP* genes was investigated in the RNAi plants. In the previous studies [28,29,31], tobacco *ZIP* genes were divided into four categories by Zn/Cd dependent expression pattern: (i) the highest expression in the apical parts (*NtZIP2, NtZIP5A*/*B, NtIRT1, NtIRT1-like*), (ii) the highest expression in the basal parts (*NtZIP1-like, NtZIP8*), (iii) uniform expression in three root parts (*NtZIP4A*/*B*), and (iv) no root-part-specific pattern (*NtZIP5-like, NtZIP11*). Of these genes, here we identified only a few whose expression levels in RNAi plants have been altered in the middle and basal root parts. The first is *NtZIP1-like* with an increased transcript level in the middle part (Figure 8f). It has already been identified as a candidate gene regulating Cd-dependent stimulation of Zn translocation [28]. NtZIP1-like is a Zn transporter strongly upregulated in the root cortex by Zn-deficiency [40]. It can be assumed that suppression of *NtZIP4A*/*B* (encoding Zn/Cd uptake transporter) might lower Zn availability in the symplast, inducing Zn-deficiency status in RNAi plants. Here, enhanced expression of *NtZIP1-like* detected in RNAi plants might complement reduced Zn uptake by cells due to the suppression of *NtZIP4*. The second was *NtZIP2*, downregulated in the middle part (Figure 8a). The physiological function of ZIP2 in plants remains largely unknown. In tobacco, it was only shown to be specifically expressed in the roots and not in leaves [28,31]. In other plant species, for example in *A. thaliana*, it was also expressed strongly in the roots [39,41], and in *Medicago truncatula*, *ZIP2* transcriptional activity in the roots was enhanced by elevated Zn [42]. Here, the detected downregulation of *NtZIP2* in the middle or basal root part of RNAi plants (Figure 8a), is in line with the suggestion that suppression of Zn-uptake transporter *NtZIP4A*/*B* induces Zn-deficiency status. Cloning and characterization of *NtZIP2* will help identify possible metabolic pathways in which both proteins participate.

Altogether, our results indicate that the responses of the examined tobacco *ZIP* genes in the middle and basal root parts to suppression of *NtZIP4A*/*B* are specific to varying degrees for distinct Zn/Cd statuses. Thus, in the root parts of RNAi plants, they might contribute to different extents to changes in Zn accumulation and cell/tissue-specific localization (primarily in the presence of Cd) and consequently to reduction of Zn translocation (Figure 1f). Confirmation of these conclusions also comes from experiments with the use of ^65^Zn. Administration of Zn locally to the basal root parts showed slightly lower amounts of ^65^Zn transferred to the shoots of RNAi plants compared with the wild-type (Figure 9).

The roles of the middle and the basal root parts in the regulation of the efficiency of metals’ root-to-shoot translocation have not been taken into consideration in studies to date. The majority of research on the understanding of mechanisms controlling this phenomenon has been performed on whole roots, and more specifically on the absorption zone within the distal root part. It has been found in several plant species, including tobacco, that the expression level of *HMA4* is a decisive factor responsible for the rate of Zn and Cd translocation [21,22]. Therefore, in our study, the expression pattern of *NtHMAα*/*β* was investigated (Supplementary File Figure S4). Interestingly, its expression level in all three root parts remained at similar level at each tested medium composition, with high upregulation at 1 µM Zn compared with Zn deficiency conditions. There are important implications of these results. First, *NtHMAα*/*β* contributes to loading of Zn and Cd to xylem vessels equally in the apical, middle, and the basal root parts; thus, ^65^Zn administered to the basal part has been taken up there and likely by *NtHMAα*/*β* loaded to xylem vessels, then transferred to the shoot. These results highlight the role of the basal root part not only in Zn storage, but also suggest participation in uptake and xylem loading. Furthermore, the presence of Cd did not modify *NtHMAα*/*β* transcript abundance, which suggested that *NtHMAα*/*β* did not contribute to the stimulation of Zn translocation in the presence of Cd.

Changes were also noted in the apical part. Compared to the wild-type, in RNAi plants, the strength of the Zinpyr-1-based green signal was higher at the proximal end of the apical meristematic region of roots from plants grown at 1 µM Zn regardless of the presence of Cd (Figure 4(a15–a18)). From the study on tobacco expressing *NtZIP4Bp::GUS*, it is known that this is where the *NtZIP4A*/*B* transcript levels were high in plants cultivated at that medium composition (Figure 5(c5,c6),(d5,d6)). Therefore, it can be assumed that in RNAi plants, the decreased expression of *NtZIP4A*/*B* just in that site contributed to modified Zn localization. Increased expression of Zn uptake genes such as *NtIRT1, NtIRT1-like*, and *NtZIP5-like* in RNAi plants (Figure 7d,e,h and Figure 8d,e,h) accompanied the specific pattern of Zn localization in the apical region (Figure 4(a15–a18)). The precise function of *NtIRT1, NtIRT1-like*, and *NtZIP5-like* in tobacco is not yet known. *NtIRT1* is upregulated in roots by low Fe, but also by Cd [28,43,44]. Induction by low Zn was shown for *NtIRT1-like* and to a lesser extent for *NtIRT1* [29]. The important finding was showing that these three genes were regulated specifically by combinations of high and low Zn and Cd concentrations. For example, *NtIRT1* in the apical root part was downregulated by 0.25 µM Cd in the Zn-deficient medium but upregulated by 1 µM Cd in the medium containing 1 µM Zn [28]. The current study points to their role in restoring the ionic balance disturbed by suppression of *NtZIP4A*/*B* and thus to the generation of the mutant’s phenotype.

In turn, the lack of influence of *NtZIP4A*/*B* suppression on Zn translocation efficiency in Zn-deficient plants regardless of the presence of Cd (Figure 1c) can be explained by the fact that Zn deficiency upregulates many genes encoding metal transporters, which due to their functional redundancy [39] may compensate for reduced expression of *NtZIP4A*/*B*. Such a function can be performed by *NtZIP1-like* and *NtZIP5A*/*B*. They encode plasma-membrane-localized Zn uptake proteins upregulated by Zn deficiency, in the roots responsible for Zn uptake by cortex and stele cells [28,40]. Here, their expression in wild-type and RNAi plants grown at 0 Zn was already very high, approximately five- to 10-fold higher compared with 1 µM Zn (Figure 7b,c vs. Figure 8b,c). Another candidate gene is *NtIRT1*, upregulated by Zn deficit [29], here found to be upregulated in the apical and middle root parts of RNAi plants exposed to 0 Zn (Figure 7d).

### 3.2. Suppression of NtZIP4A/B Decreases Cd Translocation to Shoots in Zn-Deficient Medium Supplemented with 0.25 µM Cd

Suppression of *NtZIP4A*/*B* also changed Cd root/shoot distribution, but only in plants grown in Zn-deficient medium supplemented with 0.25 µM Cd. The cadmium concentration in the roots of RNAi plants was higher than in wild-types (Figure 2b), and the Translocation Factor was reduced (Figure 2c). It is thought that in plants with reduced expression of *NtZIP4A*/*B*, similarly as with Zn, limited radial Cd transport might also occur, which could lead to increased retention of this metal in the middle and basal root parts (Figure 3) and to a lower Translocation Factor (Figure 2c). Cadmium is chemically similar to Zn. It enters plant cells through transport systems for micronutrients, primarily Zn, but also Fe, Mg, and Ca [34,45,46,47]. The NtZIP4B as a Zn and Cd transporter [31] constitutes an important route for Cd influx into the cell. Thus, its suppression might have reduced Cd transport to the symplast of root cells, including the endodermis, which may result in retention of Cd in the root cortex, reduction of radial transport, and in consequence reduced translocation to the shoot. These were accompanied in the middle root part by reduced expression of *NtZIP2* in RNAi plants (Figure 7a). Its cloning and characterization will provide new information necessary for learning more about the response of the metal homeostasis network to suppression of *NtZIP4A*/*B* and for understanding their contribution to the generation of the mutant phenotype, including alteration of the translocation of Zn and Cd.

Suppression of *NtZIP4A*/*B* only slightly lowered (insignificantly) Cd root-to-shoot distribution in plants grown at a higher Zn/Cd level (1 µM) (Figure 2f). It is likely that at a high Zn in the medium, due to competition for binding sites, NtZIP4A/B do not represent a significant pathway of Cd entry; therefore, reduction of their transcript levels did not affect Cd root/shoot distribution.

In conclusion, experiments performed on *NtZIP4A*/*B*-RNAi plants indicate that *NtZIP4A*/*B* play an important role in the control of Zn and Cd translocation to shoots in a Zn/Cd-status-dependent manner. It is noteworthy that they contribute specifically to the regulation of Cd-dependent stimulation of Zn transfer to shoots. Our results indicate that the middle and basal parts of the root act as a reservoir of excess metal in plants growing on a metal-enriched medium (Figure 3). Taking into account that *NtZIP4B*, which encodes the Zn and Cd uptake protein [31], is expressed primarily in the cortex and stele of the middle and basal root parts (Figure 5), it is hypothesized that the encoded protein could be one of the elements contributing to the regulation of the symplastic radial transport of Zn and Cd, by loading both metals into the symplast, and to the limited symplastic route of ions between the cortex cells and also across the endodermis. These processes might account for increased metal accumulation in the middle and basal root parts, as a consequence for lower root-to-shoot translocation. Symplastic radial transfer of metals present inside a cell requires their effective transport into the ER lumen. In *Arabidopsis*, such a function for Zn has been ascribed to AtMTP2 [48]; however, in tobacco the genes involved in this process are not yet known.

Furthermore, addressing the complexity of processes involved in the regulation of Zn/Cd status-dependent efficiency of Zn and Cd root-to-shoot flux, we report here several candidate target *ZIP* genes whose expression has been altered in the roots of RNAi plants, which likely due to their redundant function contribute to this phenomenon. Expression studies indicated genes that interact with *NtZIP4A*/*B* in fulfilling their physiological function in the root, including in regulating Zn and Cd translocation to the shoot. A summary of the modification of *ZIP* gene expression in RNAi plant roots is provided in Supplementary Figure S5. To fully understand the underlying processes, it is necessary also to learn about other metal homeostasis genes whose Zn/Cd status-dependent expression takes place specifically in the tissues and parts of the root, where they perform complementary functions.

Another important achievement of this study is the results of the research based on *NtZIP4B*_prom_::GUS analysis. Loss of *NtZIP4B* promoter activity in the epidermis of the middle part of the root exposed to enhanced concentration of Zn and in the presence of Cd, as well as a decrease in the cortex, indicate a protective role against metal toxicity.

## 4. Methods

### 4.1. Plant Material and General Growth Conditions

Experiments were performed on tobacco (*Nicotiana tabacum* v. Xanthi) and on RNAi *NtZIP4A*/*B* plants (generated in this study), cultivated in a controlled growth chamber at temperature 23/16 °C day/night, 40–50% humidity, 16 h photoperiod, and quantum flux density (photosynthetically active radiation (PAR)) 250 mmol m^−2^ s^−1^, fluorescent Flora tubes [31]. Tobacco seeds were obtained from the stock of the Institute of Biochemistry and Biophysics PAS, Warszawa, Poland in 2002; since then, they have been propagated in the greenhouse of the University of Warsaw for our experiments. Seeds were surface sterilized (8% sodium hypochlorite *w*/*v*, 2 min), then germinated and grown for three weeks on vertically positioned Petri dishes containing basic control medium (quarter strength Knop’s), 2% sucrose *w*/*v*, and 1% agar *w*/*v*. Next, seedlings were hydroponically grown in 1.2 L pots (two plants in each). For the first three days, they were cultivated in the control medium, then for 17 days in the quarter strength Knop’s medium with following modifications: (i) 0 Zn (Zn was not added to the medium); (ii) 0Zn + 0.25 µM Cd (as CdCl_2_); (iii) 1 µM Zn (as ZnSO_4_); (iv) 1 µM Zn + 1 µM Cd. The nutrient solution was renewed every two days. At the end of each experiment, depending on the aim, different plant parts were collected: either whole roots and shoots, or root parts, as described in detail by Palusińska et al. [28]. Briefly, three root parts were collected: (i) apical parts (2.5 cm) from the main and lateral roots, (ii) basal parts equal to 1/4 of the total length of the main roots, and (iii) middle parts containing the remaining parts of the main roots. Collected plant samples were prepared for examinations according to a procedure specific for a given experiment (details in the sections below).

### 4.2. Generation of NtZIP4A/B RNAi Plants

pENTR-TOPO-NtZIP4B-STOP plasmid [31] was used as a template. A 274 bp fragment of *NtZIP4B* cDNA was PCR amplified (named ZIP4B-delta3) using the following primers: ZIP4B-ORF-START and ZIP4B-delta3-KpnI-ClaI (sequences in the Table 1). Obtained DNA was cloned into the pENTR TM/D-TOPO^®^ vector (Invitrogen, Carlsbad, CA, USA). The correct sequence of the insert was confirmed by sequencing (Genomed, Warsaw, Poland). Using the Gateway LR Clonase enzyme mix (Invitrogen) pENTR/D-TOPO-ZIP4B-delta3 construct was recombined with pK7GWIWG2(I) plasmid [49]. The sequence was checked by sequencing. The generated plasmid was used for *N. tabacum* transformation by standard procedure of *Agrobacterium tumefaciens*, mediated as described previously [50]. Transgenic plants were selected for kanamycin resistance. The presence of RNAi cassette in T0 plants was confirmed by PCR (primers listed in Table 1). T1 seeds collected from T0 plants were germinated on Petri dishes containing a medium supplemented with 100 µM kanamycin. Eight lines with the segregation ratio 3:1 (kanamycin^toler^: kanamycin^sens^), indicating a single-locus insertion of the transgene, were used to obtain T2 homozygous lines.

### 4.3. Experiments to Investigate the Expression Pattern

There were two types of experiments. First, the transcript levels of *NtZIP4A* and *NtZIP4B* were determined in generated *NtZIP4*-RNAi lines with the segregation ratio of the transgene 3:1 (tolerant^kan^: sensitive^kan^), and compared to that of the wild-type. Whole roots and leaves from plants grown at control medium and at Zn deficiency for 17 days were collected, frozen in liquid nitrogen, and stored in −80 °C until RNA isolation.

Second, determination of the expression level of tobacco *ZIP* genes (*NtIRT1, NtIRT1-like, NtZIP1-like, NtZIP2, NtZIP4A, NtZIP4B, NtZIP5A, NtZIP5B, NtZIP5-like, NtZIP8*, and *NtZIP11*) and *NtHMAα*/*β* in the apical, middle, and basal root parts from the wild-type plants and from the selected RNAi lines, cultivated in four variants of medium composition (details in the Section 4.1). Experiments were performed in three replicates. For each, material was collected from twelve plants.

Collected plant parts were frozen in liquid nitrogen and stored in −80 °C until expression analysis. Extraction of total mRNA (Plant RNA Mini Kit, Syngen, Wrocław, Poland) and subsequent RT-qPCR reaction in a Roche mastercycler (LightCycler^®^480 System, Roche, Diagnostics, Rotkreuz, Switzerland) using Light Cycler480 SYBR Green (Master 0488735001) were performed according to the manufacturer’s recommendations. Details were described previously by Barabasz et al. [31]. Briefly, the comparative ΔCt (threshold cycle) method was used to calculate the relative quantities of each transcript in the samples [51]. As an internal control tobacco *NtPP2A* (protein phosphatase 2A; AJ007496) was used, and its stability in the range of applied metal concentrations are given in Supplementary Figure S7. Primer sequences are listed in Table 1.

### 4.4. Determination of Zn and Cd Concentrations

At the end of 17-day exposure of the wild-type and *NtZIP4B*-RNAi plants to tested combinations of Zn and Cd concentrations (see Section 4.2), roots were washed briefly in distilled water, then in 5 mM CaCl_2_ at 4 °C for 15 min, and again in water. The method of collecting plant samples depended on the purpose of the experiment.

To determine the efficiency of the root-to-shoot translocation of Zn and Cd, roots and shoots were separated and dried at 55 °C in an oven and dry biomass was measured. Based on established concentrations of Zn and Cd, the Translocation Factor (TF) was calculated as the ratio of shoot/root Zn and Cd concentrations [29].

To determine concentrations of Zn and Cd in the root parts, the main roots were cut into the apical, middle, and basal parts (see Section 4.2) and dried in 55 °C. Dried plant samples were acid digested in 65% HNO^3^ and 39% H_2_O_2_ (9:1, *v:v*) in a closed system microwave mineralizer (Milestone Ethos 900, Milestone, Bergamo, Italy). Concentrations of Zn and Cd were measured by flame atomic absorption spectrophotometry (TJA Solution Solar M, Thermo Electron Manufacturer Ltd., Cambridge, UK) [50,52]. Certified reference material (Virginia tobacco leaves CTA-VTL-2; Commission for Trace Analysis of the Committee for Analytical Chemistry PAS and Institute of Nuclear Chemistry and Technology, Warsaw) was included in each analysis run.

### 4.5. GUS-Based Analysis of NtZIP4B Spatial Expression in Root Parts

To determine whether the tissue-specific expression of *NtZIP4B* in the apical, middle, and basal root parts changes following 17-day exposure to Zn deficiency or to 1 μM Zn with and without Cd (0.25 and 1 μM Cd), tobacco plants expressing *pMDC163::NtZIP4Bp::GUS*, generated previously [31] were used for analysis (two homozygous lines numbers 1-8-3; 2-18-1). Whole roots were subjected to GUS histochemical staining as described by Barabasz et al. [31]. At the end, roots were additionally fixed in FAA (50% ethanol; 5% formaldehyde; 10% acetic acid) for 30 min and cleared with increasing concentrations of ethanol (50%, 70%). Then the apical, middle, and basal root fragments were dehydrated in an alcohol series and embedded in Technovit 7100 resin. Hand-made sections were analyzed under the microscope (OPTA-TECH, Warsaw, Poland).

### 4.6. Determination of Zn Localization in Root Parts

Zn localisation was determined in the apical, middle, and basal parts of the roots from wild-type and RNAi *NtZIP4B* plants (two homozygous lines; numbers 4 and 6) exposed for 17 days to tested combinations of Zn and Cd concentrations in the medium (0 Zn; 0Zn + 0.25 μM Cd; 1 μM Zn; 1 μM Zn + 1 μM Cd). Roots were cut into parts (see Section 4.1), embedded in 3% agarose, and cut longitudinally into 150 μm sections on a Vibratome (Leica Biosystems VT1000S, Nussloch, Germany). They were stained with 10 μM Zinpyr-1 (Sigma) in 0.9% NaCl for 1 h in the dark and subsequently washed in 0.9% NaCl as described by Siemianowski et al. [53]. The fluorescent signal of Zinpyr-1-bound Zn was monitored under the fluorescent microscope (OPTA-Tech) and an inverted Leica TCS SP2 confocal microscope (Leica Microsystems Inc., Wetzlar, Germany) using excitation at 470 ± 20 nm and detection at 525 ± 25 nm.

### 4.7. ^65^Zn Radiolabeling Experiments

To investigate contribution of the basal root parts in Cd-dependent uptake and translocation of Zn to shoots and evaluate the role of *NtZIP4A*/*B* in this process, wild-type and two transgenic lines (numbers 4 and 6) of *NtZIP4A*/*B* RNAi plants were used for experiments. The 3.5-week-old plants were further grown in the liquid ¼ Knop’s medium supplemented with 1 μM Zn, with or without the presence of 1 μM Cd, for six days in 2.4 L pots.

On the last, seventh day of exposure, plants were transferred to Petri dishes containing 250 mL of the agar solidified (0.8%) nutrient medium covered with 10 mL of the liquid medium of the same composition. Plants were placed horizontally with a support for the shoots (so that they were not in contact with the medium).

The basal root parts of each experimental plant were immersed in a small vial containing 2 mL of the medium of the same composition but with 1 μM of radioactive ^65^Zn. The ^65^Zn was obtained by activating the ZnSO_4_ in National Center for Nuclear Research in Świerk (Warsaw, Poland). At the beginning, the medium in small vials containing ^65^Zn was replaced with the fresh one three times every 2 h. Exposure lasted 24 h.

At the end of the experiment, shoots were cut out from the roots, pooled within each experimental variant (six plants per each variant), and dried. The level of radioactive ^65^Zn in dried shoots was determined by gamma spectrometer with an coaxial HPGe detector (Canberra Packard, Warsaw, Poland). The experiment was performed in three biological replicates.

### 4.8. Statistical Analysis

Evaluation of the statistical significance was performed at the 0.05 or 0.1 probability level using Student’s *t*-test. Experiments were performed with at least three independent replicates.

## Figures and Tables

**Table 1:** Primers sequences used in the study.

Gene Name/Target	Primer For	Primer Rev	Products on Target Templates	Length of Amplicon
**Primers for qPCR**				
*NtIRT1*	CGCAATAACAACTCCATTCG	AAGCCATATAGATCAGAAGGC	AB263746.1	134
*NtIRT1-like*	CTTCTTCGCAGTAACAACC	AGCCATGTAAATAAGAAGACC	XM_016611068.1	139
*NtZIP1-like*	TGCTGCTGGTGTCATTCTAG	GCTGGCATGACTATGACTGTG	XM_016652513.1	274
*NtZIP2*	CACCATGTTTAGTGACTGC	CTTGAGAAAAGGATTTGCTTCC	XM_016617597.1	136
*NtZIP4A*	CTGTTTCCAATACCACCTGT	GCTTCTTGCCAACTAATGGA	XM_016647965.1	150
*NtZIP4B*	ACTGACTCTAATCTCTTTCTTGC	ATGGCGATAAAACCAGCG	XM_016586154.1	161
*NtZIP5A*	TGGGAACTCTTATGGTGGAT	CTGAACCATGTGAATGAACATGA	NM_001325745.1	126
*NtZIP5B*	GGGAACTCTAATGGTGGAC	ATGTGCATGGCCATGTG	XM_016593570.1	130
*NtZIP5-like*	TCTGCGAAAAATGGTGTG	GAAGGAGCTCGGAATCAG	XM_016594002.1	118
*NtZIP8*	GGTTGTGCCATTAGAAGAGG	AGTTAATGCCGCTACAAGG	XM_016603305.1, XM_016586286.1	163
*NtZIP11*	CTGACACAGATTCCGACTCA	CACAATCAGCCAACATAGTAAGC	XM_016644574.1	321
*NtHMAα*/*NtHMAβ*	TCCTCAAACGTGCTCTACC	TGGAGCTTGAAGTTGCAGA	HF675181.1, HF937054.1	188
*NtPP2A*	GCACATTCATTCAGTTTGAACC	GTAGCATATAAAGCAGTCAGC	NM_001325282.1	142
**Primers used for amplification of *NtZIP4B-delta3* fragment**				
5′-terminal fragment of NtZIP4B coding sequence	CACCATGTCGTTCACTGAGGATCTCGTGCCC (named ZIP4B-ORF-START)	AGTCAGATCGATGGTACCGATGCTTCTTGC (named ZIP4B-delta3-KnpI-ClaI)	Plasmid pENTR-TOPO-NtZIP4B-STOP	274
**Primer used for confirmation of RNAi cassette in T_0_ plants**				
NtZIP4B-delta3 in ‘sens” orientation	CTATCCTTCGCAAGACCCTTC	CATAACTCAGCACACCAGAG	Plasmid pK7GWIWG2-ZIP4B-delta3	662
NtZIP4B-delta3 in “antisens” orientation	CTTCTTAGCATTTAACGTGTTTGC	CCTTATCTGGGAACTACTCAC	Plasmid pK7GWIWG2-ZIP4B-delta3	535

## Supplementary Materials

The following are available online at https://www.mdpi.com/article/10.3390/ijms22105355/s1.

## Abbreviations

Cd.	Cadmium
GUS	β-glucuronidase
RNAi	RNA interference
ZIP	ZRT-IRT-like Proteins
Zn	Zinc
