# Supplementary material for: Suppression of NtZIP4A/B Changes Zn and Cd Root-to-Shoot Translocation in a Zn/Cd Status-Dependent Manner
Source: Int J Mol Sci. 2021 May 19;22(10):5355. doi: 10.3390/ijms22105355 (PMC8161331; doi:10.3390/ijms22105355)
Supplement: Supplementary file 1 [file ijms-22-05355-s001.zip › Supplementary Figure S1.pdf]

Supplementary Figure S1: Generation and characteristics of *NtZIP4*-RNAi plants

Content:

A. Construct……………………………………………………………………….…1

B. Results of transformation …………………………………………………….….2

C. Suppression of *NtZIP4A/B* by RNA interference (RNAi): expression level of *NtZIP4A* and *NtZIP4B* in homozygous lines …………………………………4

D. Development of T2 homozygous lines throughout the whole life cycle (soil experiments) ………………………………………………………...…5

A. Construct

p*ENTR-TOPO-NtZIP4B-STOP* plasmid [1] was used as a template.
A 274 bp fragment of *NtZIP4B* cDNA was amplified (named ZIP4B-delta3) using primers named ZIP4B-ORF-START and ZIP4B-delta3-KpnI-ClaI (sequences are given below). Obtained DNA was cloned into *pENTR*^{TM}*/D-TOPO*$^{®}$ vector. The correct sequence of the insert was confirmed by sequencing (Genomed, Poland). *pENTR/D-TOPO-ZIP4B-delta3* construct was recombined with *pK7GWIWG2(I)* plasmid [2] using LR clonase. The sequence was checked by endonuclease digestion and sequencing. Generated plasmid was used for *N. tabacum* transformation by standard *Agrobacterium tumefaciens*-mediated procedure.

Primers:

```
ZIP4B-ORF-START: CACCATGTCGTTCACTGAGGATCTCGTGCCC

ZIP4B-delta3-KpnI-ClaI: AGTCAGATCGATGGTACCGATGCTTCTTGC
```

Alignment between the 5'-terminal 360 bp fragments of *ZIP4A* and *ZIP4B* genes and *ZIP4B-delta3* sequence is shown below:

In yellow –marked nucleotides from *ZIP4B* sequence different from *ZIP4A* sequence,

In red - KpnI restriction site generated by a primer

In blue – ClaI restriction site generated by a primer

```
ZIP4B_ORF       ATGTCGTTCACTGAGGATCTCGTGCCCTTCTTTTTTATGGACCCAAAAATTAGAGAAAAG     60
ZIP4A_ORF       ATGTCGTTCATTGAGGATCTCGTGCCCTTCTTTTTTATGGACCCAAAATCTAGAGAAAAG     60
ZIP4B-delta3    ATGTCGTTCATTGAGGATCTCGTGCCCTTCTTTTTTATGGACCCAAAATCTAGAGAAAAG     60
                ********** ************************************  **********

ZIP4B_ORF       ACCGGGGCTTTCTCAGATACCGTTATGCTGAAACTTTATCAATCTGTTTCCAATATTACC     120
ZIP4A_ORF       ACTGGGGCTTTCTCAGATACCATTATGCTGAAACTTTATCAATCTGTTTCCAATACCACC     120
ZIP4B-delta3    ACTGGGGCTTTCTCAGATACCATTATGCTGAAACTTTATCAATCTGTTTCCAATACCACC     120
                ** ****************** *********************************  ***

ZIP4B_ORF       TGCGGCAGTGCTGATGAAGAGATAGAAGGCTGCCGAGACAGCTCGGCTGCTCTTACCCTT     180
ZIP4A_ORF       TGTGGCAGTGCTGATGAAGAAATAGAAGGCTGCCGAGACAGCTCGGCTGCTCTCACCCTT     180
ZIP4B-delta3    TGTGGCAGTGCTGATGAAGAAATAGAAGGCTGCCGAGACAGCTCGGCTGCTCTCACCCTT     180
                ** ***************** ******************************** ******

ZIP4B_ORF       AAAATCGTGGCTATCTCTGCCATCCTAATAGCTAGTACTTGCGGAGTTGGTATCCCGTTA     240
ZIP4A_ORF       AAAATTGTGGCTATCTCTGCCATCCTCATAGCTAGCACTTGCGGAGTTGGTATTCCATTA     240
ZIP4B-delta3    AAAATTGTGGCTATCTCTGCCATCCTCATAGCTAGCACTTGCGGAGTTGGTATTCCATTA     240
                ***** ******************** ******** ***************** ** ***

ZIP4B_ORF       GTTGGCAAGAAGCATCGGTTCCTCCGAACTGACTCTAATCTCTTTCTTGCTGTTAAAGCC     300
ZIP4A_ORF       GTTGGCAAGAAGCATCGGTTCCTCCGAACTGACTCCAATCTCTTTCTTACTGTTAAAGCC     300
ZIP4B-delta3    GTTGGCAAGAAGCATCGGTaCCatCGAtCTGACT--------------------------     274
                ******************* **  *** ******

ZIP4B_ORF       TTTGCTGCTGGTGTCATCCTCTCTACAGGCTTTGTCCACATATTACCAGGCGCCACCTCA     360
ZIP4A_ORF       TTTGCTGCTGGTGTCATCCTCTCTACAGGATTTGTCCACATATTACCAGGCGCCACCTCA     360
ZIP4B-delta3    ------------------------------------------------------------     274
```

(B) Results of transformation

28 transgenic plants were regenerated. Out of them:

- 4 plants died after planting into the soil
- in 4 plants the presence of T-DNA was not confirmed by PCR
- 20 lines with confirmed T-DNA (by PCR) were derived,

for 12 lines the segregation ratio (kanamycin^{toler} : kanamycin^{sensit}) was 3:1 suggesting single-locus insertion of the transgene; they were marked in yellow in Table 1 below.

from these lines there were selected 8 homozygous lines (ZIP4BΔ3-k1-r1, -k1-r3, -k1-r7, -k4-r3, -k4-r7, -k4-r8, -k4-r9, -k4-r11) – marked in blue in Table 1 below.

Table 1. Summary of the generation of RNAi *NtZIP4* transgenic plants. The results of the analysis of the transgene segregation in T1 generation of RNAi NtZIP4 lines are given.

No.	Line name	Transgen confirmation by PCR	Segregation (% of kan^{toler})
1	ZIP4BΔ3-k1-r1	YES	74,86% ± 6,44% (n=10)
2	ZIP4BΔ3-k1-r2	Died in the soil	-
3	ZIP4BΔ3-k1-r3	YES	75,02% ± 4,96% (n=5)
4	ZIP4BΔ3-k1-r4	YES	69,77% ± 10,77% (n=26)
5	ZIP4BΔ3-k1-r5	YES	all plants sensitive
6	ZIP4BΔ3-k1-r6	Died in the soil	-
7	ZIP4BΔ3-k1-r7	YES	74,06% ± 6,45% (n=25)
8	ZIP4BΔ3-k1-r8	YES	all plants sensitive
9	ZIP4BΔ3-k1-r9	YES	all plants sensitive
10	ZIP4BΔ3-k1-r10	YES	92,20% ± 3,57% (n=5)
11	ZIP4BΔ3-k1-r11	YES	7,26% ± 4,42% (n=4)
12	ZIP4BΔ3-k1-r12	YES	76,53% ± 0,37% (n=3)
13	ZIP4BΔ3-k4-r1	Died in the soil	-
14	ZIP4BΔ3-k4-r2	NO	all plants sensitive
15	ZIP4BΔ3-k4-r3	YES	64,41% ± 16,47% (n=24)
16	ZIP4BΔ3-k4-r4	YES	all plants sensitive
17	ZIP4BΔ3-k4-r5	YES	74,07% ± 5,31% (n=8)
18	ZIP4BΔ3-k4-r6	NO	-
19	ZIP4BΔ3-k4-r7	YES	73,04% ± 7,34% (n=20)
20	ZIP4BΔ3-k4-r8	YES	75,78% ± 8,03% (n=20)
21	ZIP4BΔ3-k4-r9	YES	76,08% ± 5,47% (n=15)
22	ZIP4BΔ3-k4-r10	Died in the soil	-
23	ZIP4BΔ3-k4-r11	YES	71,81% ± 5,91% (n=10)
24	ZIP4BΔ3-k4-r12	YES	75,10% ± 20,43% (n=10)
25	ZIP4BΔ3-k4-r13	YES	89,53% ± 3,93% (n=3)
26	ZIP4BΔ3-k4-r14	NO	all plants sensitive
27	ZIP4BΔ3-k4-r15	NO	all plants sensitive
28	ZIP4BΔ3-k4-r16	YES	97,50% ± 2,50% (n=5)

The names of eight selected lines of T1 generation (marked in blue in the Table 1 above) are given below, and the names of T2 homozygous lines derived from them, respectively.
In these lines the level of *NtZIP4A/B* silencing was analysed.

**T1 lines**	nos of **T2 lines**
ZIP4BΔ3-k1-r1	4
ZIP4BΔ3-k1-r3	6
ZIP4BΔ3-k1-r7	7
ZIP4BΔ3-k4-r3	3
ZIP4BΔ3-k4-r7	10
ZIP4BΔ3-k4-r8	8
ZIP4BΔ3-k4-r9	9
ZIP4BΔ3-k4-r11	11

(C) Suppression of *NtZIP4A/B* by RNA interference (RNAi): expression level of *NtZIP4A* and *NtZIP4B* in homozygous lines

In the transgenic homozygous *NtZIP4A/B*-RNAi lines the expression levels of *NtZIP4A* and *NtZIP4B* were determined in the whole roots and leaves collected from 6-week-old plants (wild-type WT, and eight selected homozygous RNAi lines) hydroponically grown at control conditions (quarter-strength Knop's medium) and for the last four days exposed to Zn deficiency. Reduction of the transcript level of two *NtZIP4* genes (*NtZIP4A* and *NtZIP4B*) were observed in *NtZIP4A/B*-RNAi plants. Results are given in the Figure 1 below.

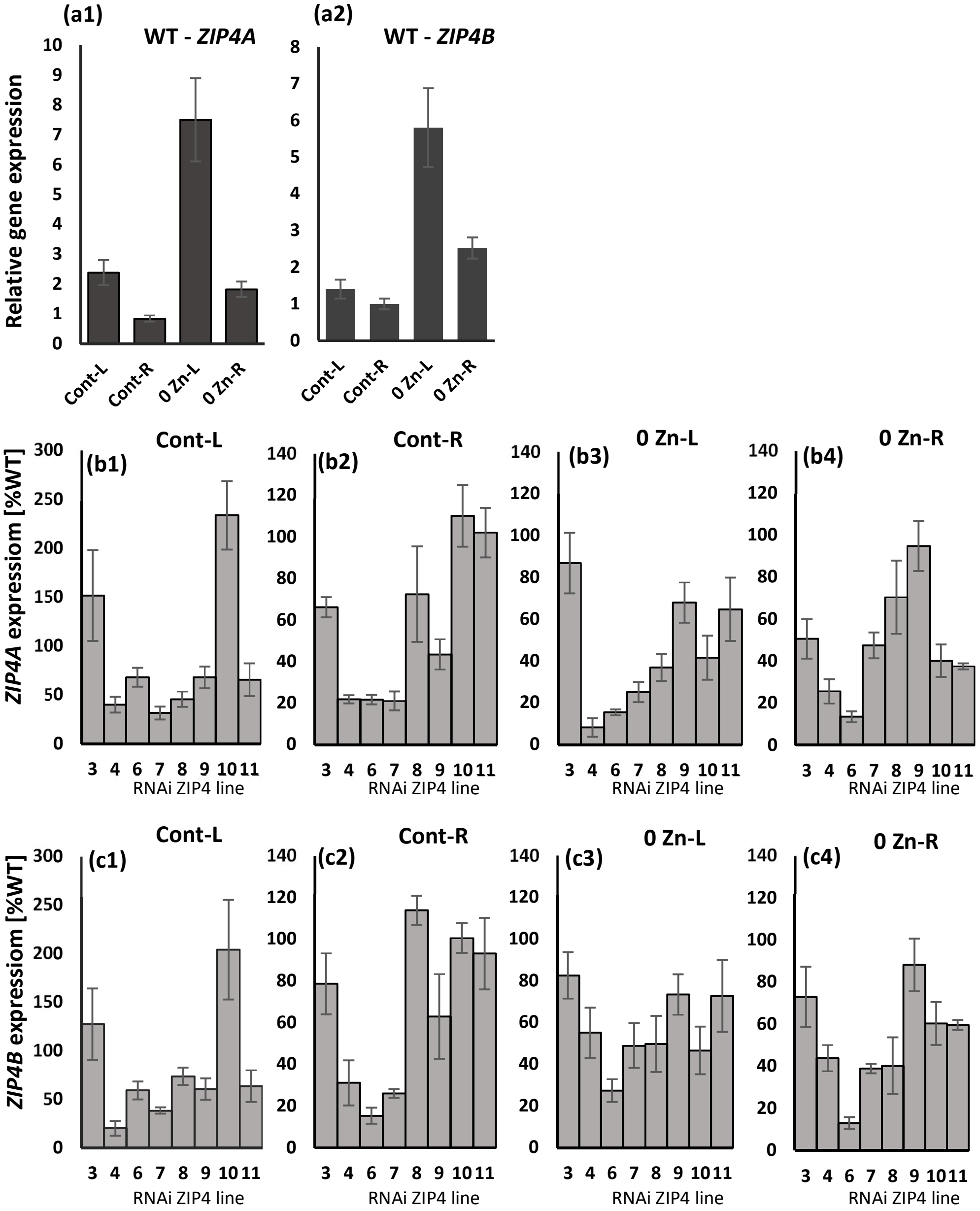

Figure 1: Normalized expression of *NtZIP4A* and *NtZIP4B* in the whole roots or leaves from plants (wild type and RNAi lines) grown under control conditions and at Zn deficit. 38 day-old plants grown in the 1/4 Knop's medium were exposed to Zn deficit for 4 days, and in parallel to the control medium. Gene expression was normalized to the *PP2A* level. Values correspond to arithmetic means ±SD (n = 3); those with the ratio greater than 2 are considered significantly different.

NtZIP4A (**a1**) and *NtZIP4B* (**a2**) expression in leaves (L) and roots (R) of WT plants cultured under control ¼ Knop's (Cont) or Zn deficiency (0 Zn) conditions.
Reduction of the *NtZIP4A/B* transcript levels in tested RNAi lines shown as the ratio of each RNAi line expression to wild-type expression (respectively for each treatment and tested organ) expressed as a percentage (**b1-b4; c1-c4**).
NtZIP4A (**b1, b2, b3, b4**) and *NtZIP4B* (**c1, c2, c3, c4**) expression in leaves (**b1, b3, c1, c3**) and roots (**b2, b4, c2, c4**) of RNAi *NtZIP4* lines under control **(b1, b2, c1, c2**) and Zn deficiency (**b3, b4, c3, c4**) conditions.

(D) Growth and development of T2 homozygous lines throughout the whole life cycle (soil experiments)

The *NtZIP4A/B*-RNAi (four homozygous T2 lines: nos 4, 6, 7, 8) and wild-type plants were grown in soil from sowing until flowering and seed harvesting. They were grown in the greenhouse with controlled conditions (photoperiod 16 h day / 8 h night, temperature 24°C ±4°C). After the appearance of flower buds, the length of the shoot and the number of leaves were measured. Following the full formation of inflorescences, the total number of flowers was counted.
The photographs below show 4-month-old plants (wild-type and T2 generation of four *NtZIP4A/B*-RNAi lines: nos 4, 6, 7 and 8).

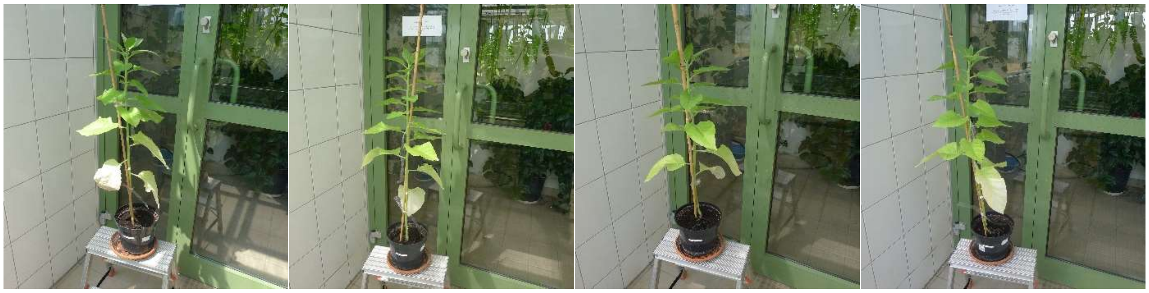

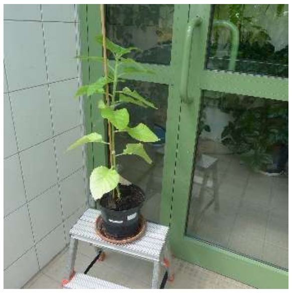

NtZIP4A/B-RNAi line no. 4

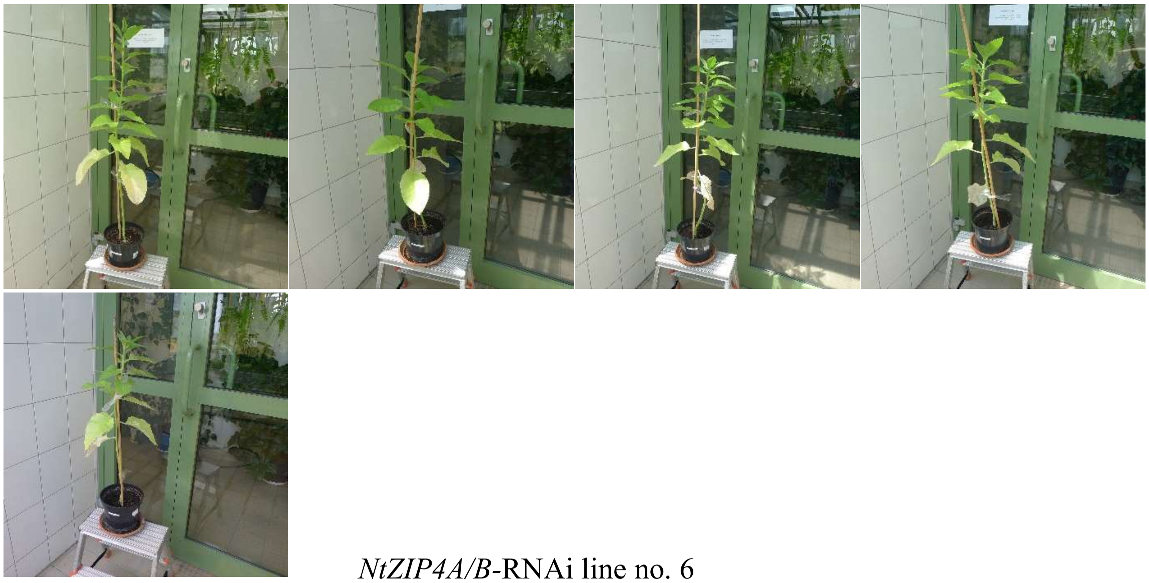

NtZIP4A/B-RNAi line no. 6

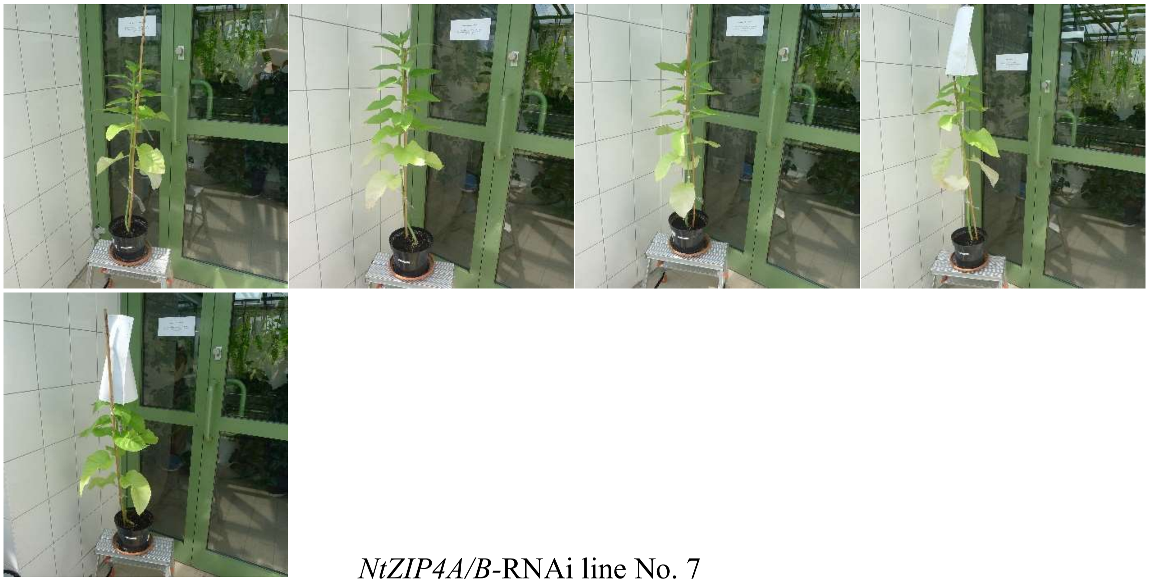

NtZIP4A/B-RNAi line No. 7

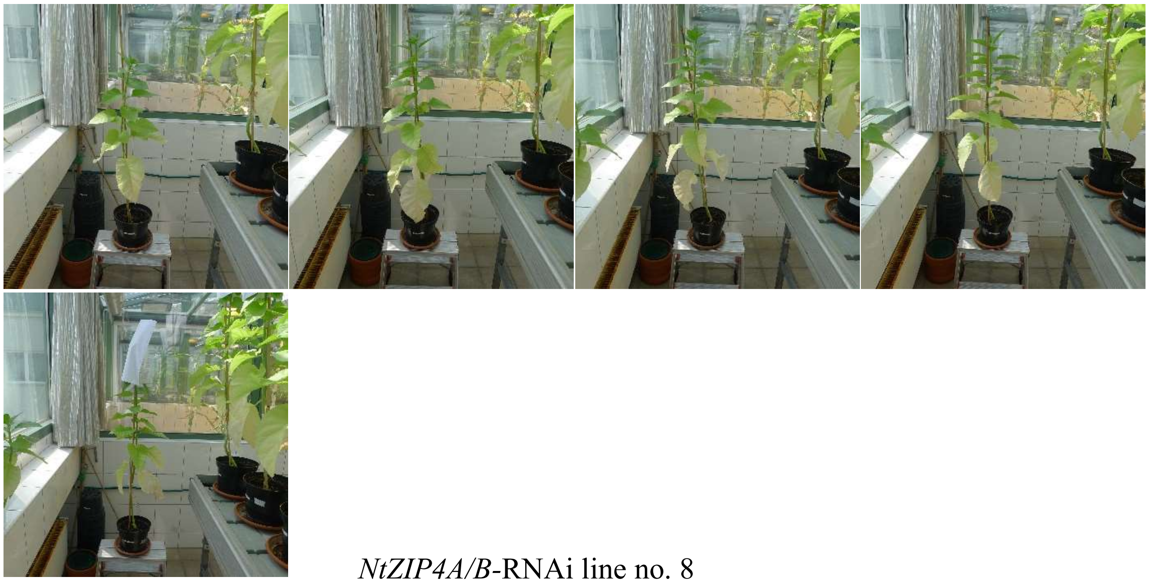

NtZIP4A/B-RNAi line no. 8

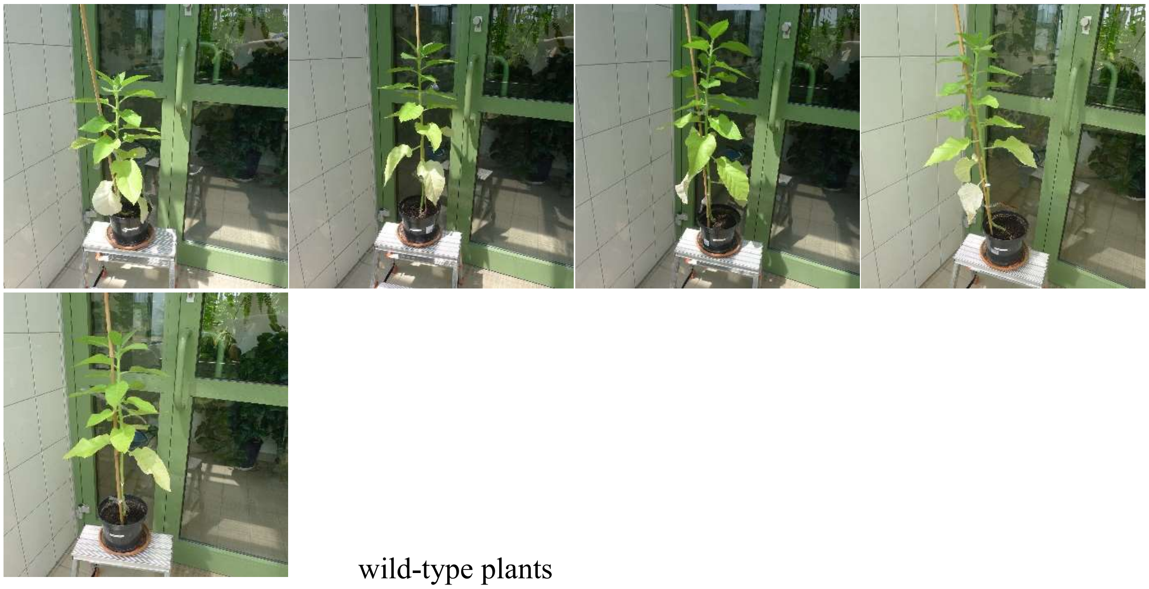

wild-type plants

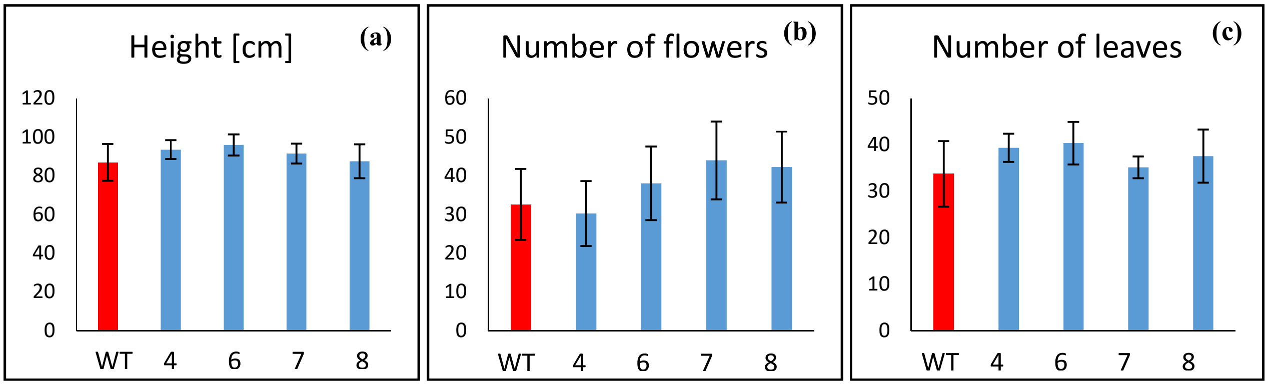

Figure 2: Developmental parameters at the beginning of flowering of four *NtZP4*-RNAi lines (nos 4; 6; 7; 8) and wild-type (WT): (a) shoot height [cm]; (b) number of flowers, (c) number of leaves in wild-type plants and in T2 generation of four *NtZIP4A/B*- RNAi lines nos 4; 6; 7 and 8.

References:

1. Barabasz, A.; Palusińska, M.; Papierniak, A.; Kendziorek, M.; Kozak, K.; Williams, L.E.; et al. Functional analysis of *NtZIP4B* and Zn status-dependent expression pattern of tobacco *ZIP* genes. *Frontiers Plant Sci*. **2019**, *9*, 1984.
2. Karimi, M.; Inzé, D.; Depicker, A. GATEWAY vectors for Agrobacterium-mediated plant transformation. *Trends Plant Sci.* **2002**, *7*, 193-5.
