# Supplementary material for: Suppression of NtZIP4A/B Changes Zn and Cd Root-to-Shoot Translocation in a Zn/Cd Status-Dependent Manner
Source: Int J Mol Sci. 2021 May 19;22(10):5355. doi: 10.3390/ijms22105355 (PMC8161331; doi:10.3390/ijms22105355)

Supplementary Figure S2:

Zn distribution at the longitudinal sections through the apical, middle and basal parts of the roots from wild-type and *NtZIP4A/B*-RNAi plants, grown at Zn deficient medium with or without 0.25 µM Cd, visualized by Zinpyr-1.

3.5-week old plants of wild-type (WT) and *NtZIP4A/B*-RNAi lines (no. 4, no. 6) grown in the quarter-strength Knop's medium, were exposed for 17 days to the Zn-deficient medium (Zn was not added to the medium) with or without 0.25 µM Cd.

Fluorescence microscope images of longitudinal sections through the apical, middle and basal root parts (a). Representative pictures of the anatomical structure of the root parts at bright field (b). Plants grown at 0 Zn (a1, a3, a5; b1, b3, b5); at 0 Zn + 0.25 µM Cd (a2, a4, a6; b2, b4, b6). Wild-type plants (WT); RNAi plant lines (Line 4, line 6).

Magnification bar = 0.25 mm.

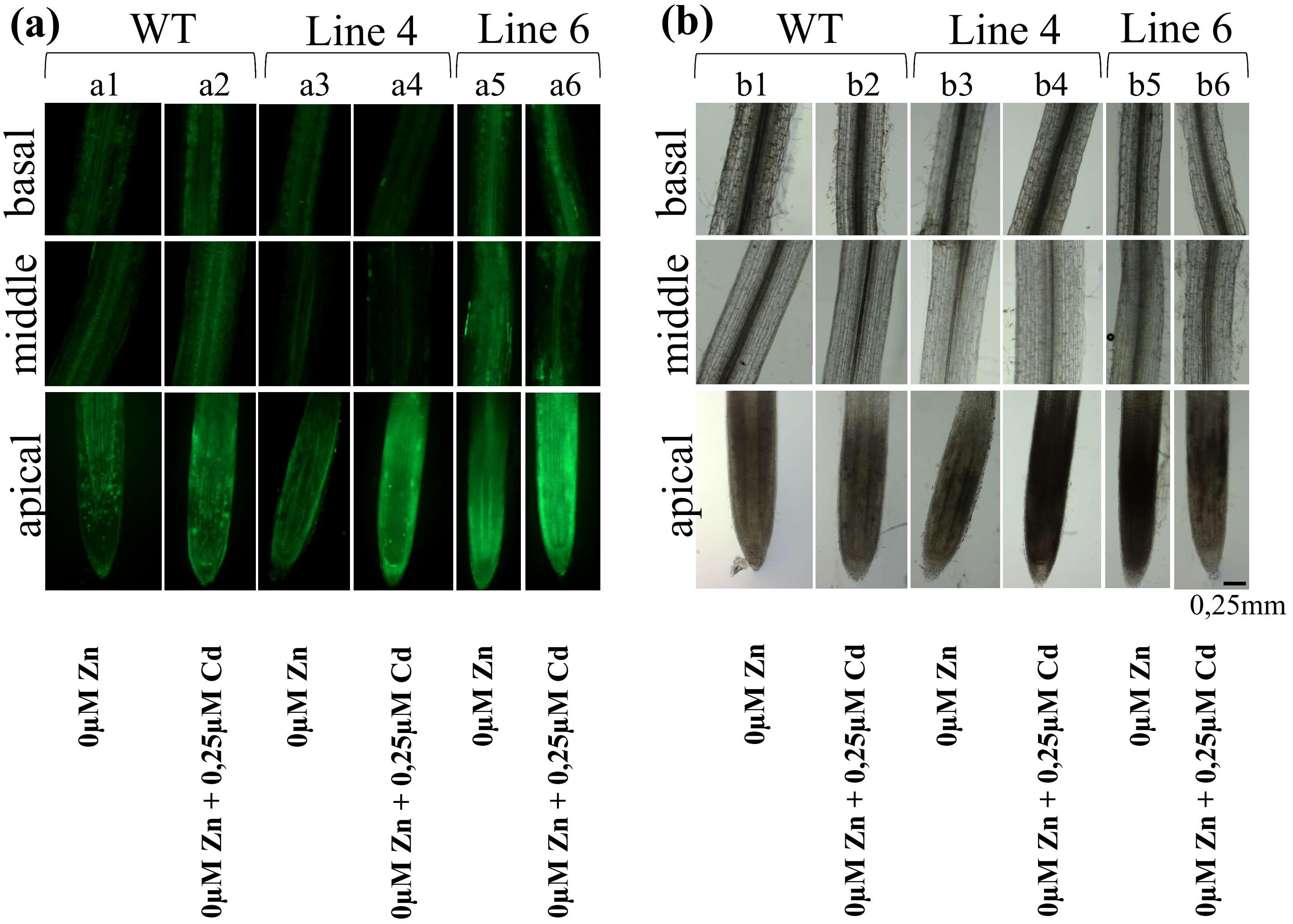

Supplement: Supplementary file 1 [file ijms-22-05355-s001.zip › Supplementary Figure S2.pdf]
