# Supplementary material for: Suppression of NtZIP4A/B Changes Zn and Cd Root-to-Shoot Translocation in a Zn/Cd Status-Dependent Manner
Source: Int J Mol Sci. 2021 May 19;22(10):5355. doi: 10.3390/ijms22105355 (PMC8161331; doi:10.3390/ijms22105355)
Supplement: Supplementary file 1 [file ijms-22-05355-s001.zip › Supplementary Figure S3.pdf]

Supplementary Figure S3: Stability of *NtPP2A*

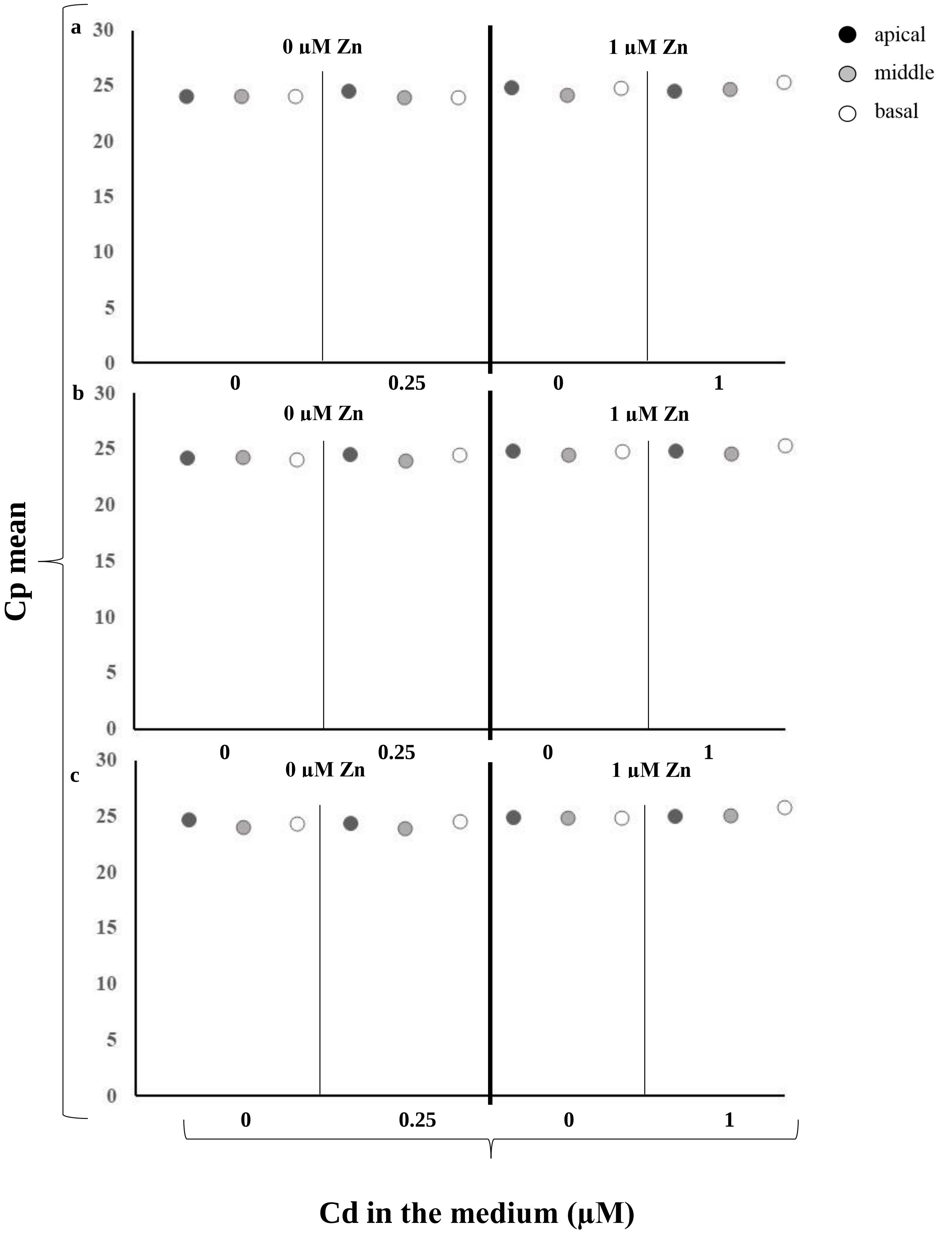

RNA transcription levels of *PP2A* gene, presented as Cp mean values in the apical, middle and basal root parts collected from: (a) wild-type plants; (b) the RNAi plants with the reduced *NtZIP4* mRNA level (line no. 4); (c) the RNAi plants with the reduced *NtZIP4* mRNA level (line no. 6). All plants were grown for 17 days on ¼ Knop's medium containing: 0 µM Zn + 0 µM Cd, 0 µM Zn + 0.25 µM Cd, 1 µM Zn + 0 µM Cd and 1 µM Zn + 1 µM Cd.
