# Supplementary material for: Suppression of NtZIP4A/B Changes Zn and Cd Root-to-Shoot Translocation in a Zn/Cd Status-Dependent Manner
Source: Int J Mol Sci. 2021 May 19;22(10):5355. doi: 10.3390/ijms22105355 (PMC8161331; doi:10.3390/ijms22105355)
Supplement: Supplementary file 1 [file ijms-22-05355-s001.zip › Supplementary Figure S4.pdf]

Supplementary Figure S4: Expression of *NtHMAα/β*

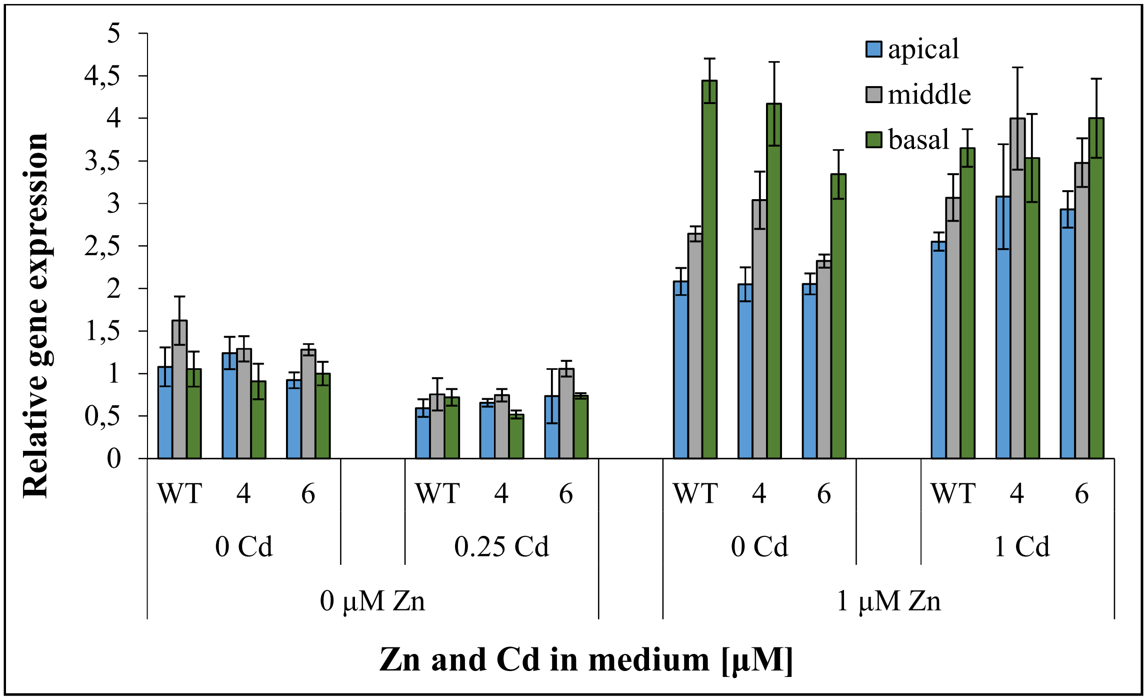

Expression of *NtHMAα/β* (primers were used which amplify both *NtHMAα* and *NtHMAβ* sequences) in the apical, middle and basal parts of the roots from plants grown under various Zn and Cd concentrations.
3.5-week-old plants grown in the control medium (quarter-strength Knop's) were exposed to the control medium supplemented with pairwise combinations of Zn (0; 1 μM) and Cd (0; 0.25; 1 μM) for 17 days. Primers were used that amplify both *NtHMAα* and *NtHMAβ* sequences. Gene expression was normalized to the *PP2A* level. Values correspond to arithmetic means ±SD (n = 3); those with the ratio greater than 2 were considered significantly different.
