# Supplementary material for: Suppression of NtZIP4A/B Changes Zn and Cd Root-to-Shoot Translocation in a Zn/Cd Status-Dependent Manner
Source: Int J Mol Sci. 2021 May 19;22(10):5355. doi: 10.3390/ijms22105355 (PMC8161331; doi:10.3390/ijms22105355)

Supplementary Figure S5:

Summary: consequences of *NtZIP4A/B* suppression on the expression of *ZIP* genes in the apical, middle and basal parts of the roots grown at four combinatins of Zn and Cd concentrations.

Changes in the expression level of tobacco *ZIP* genes in the apical, middle and basal root parts of *NtZIP4A/B*-RNAi plants relative to the wild-type. Arrows indicate increase or decrease in the expression relative to the expression in the wild-type grown at the same experimental conditions (graphical summary of the results shown in Figure 7 and Figure 8).

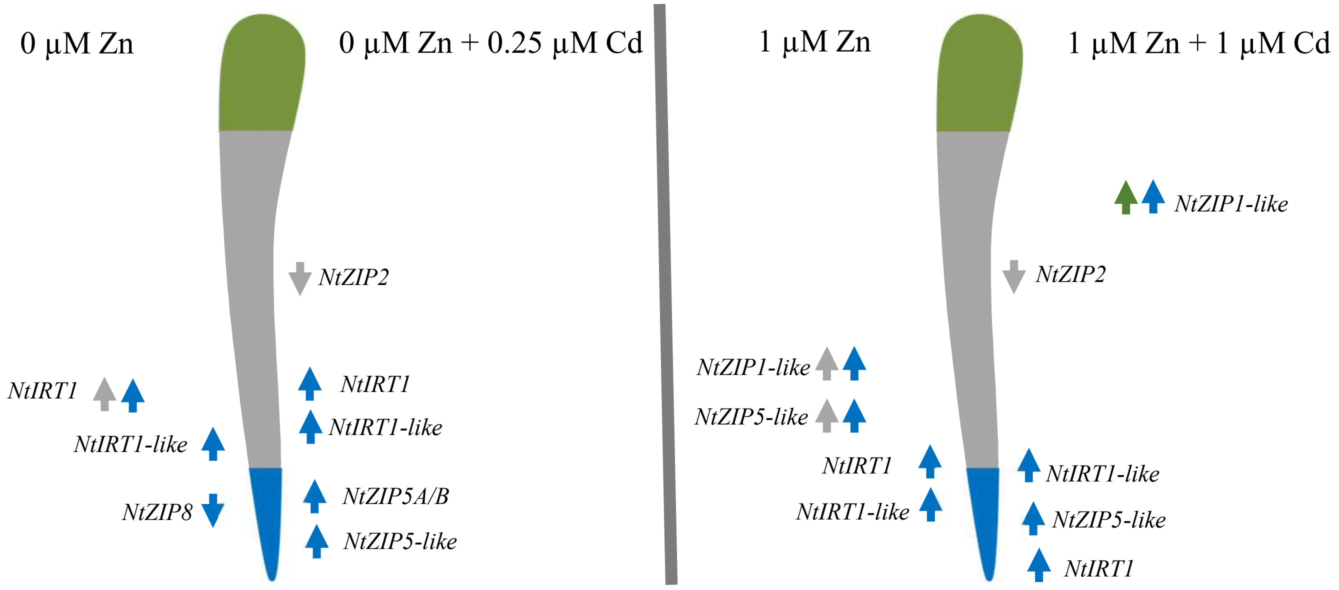

Supplement: Supplementary file 1 [file ijms-22-05355-s001.zip › Supplementary Figure S5.pdf]
